# Risk perception of electromagnetic fields among school teachers and nursery school teachers: a mixed-methods study

**DOI:** 10.1186/s12889-026-27964-3

**Published:** 2026-06-23

**Authors:** Katharina Lüthy, Claudia Riesmeyer, Jessica Kühn, Felix Forster, Katja Radon, Tobias Weinmann

**Affiliations:** 1 Institute and Clinic for Occupational, Social and Environmental Medicine, LMU Medizin, LMU University Hospital, Ludwig-Maximilians-Universität München, Munich, Germany; 2 Department of Media and Communication, Ludwig-Maximilians-Universität München, Munich, Germany

**Keywords:** Electromagnetic Fields, School teachers, Nursery school teachers, Risk Perception, Knowledge, Latent class analysis, Health communication

## Abstract

**Background:**

Although there is no evidence for adverse health effects from non-ionising electromagnetic fields (EMFs) exposure below legal limits, this concern is widespread among the general population. School teachers and nursery school teachers are considered multipliers of health-related information as they impart knowledge to parents and children. However, there is a lack of knowledge about the risk perception regarding EMFs among these professional groups. The extent to which school teachers and nursery school teachers are in contact with questions concerning EMFs and health in their work has not been investigated. Nor is there any knowledge about the information level of school teachers and nursery school teachers regarding EMFs. Data on teachers’ perception regarding EMFs is important though to meet information needs.

**Methods:**

A cross-sectional study among school teachers and nursery school teachers in Germany was carried out in 2024, combining an online survey (*N* = 1400) with focus groups (*N* = 29). We calculated prevalence estimates for participants’ risk perception, subjective information level, and EMFs relevance in everyday work with correction for non-response. Furthermore, we conducted a latent class analysis to identify types of EMFs risk perception.

**Results:**

A third of all participating school teachers (32%) and nursery school teachers (33%) indicated that, in their view, EMF exposure below legal limits may cause adverse health effects. Five types of risk perception concerning EMFs were identified using latent class analysis, with the high risk perception class comprising 11% of the participants. Many school teachers (56%) and nursery school teachers (77%) perceived themselves as poorly informed about EMFs. The group discussions provided deeper insights and supported these findings. The interviews revealed that both professional groups had concerns about the health effects of EMFs. Knowledge of scientific evidence concerning EMFs and health was low. Moreover, the discussions revealed a desire for more information on EMFs and health.

**Conclusions:**

A notable proportion of school teachers and nursery school teachers indicated considerable risk perception towards EMFs. Most participants indicated low subjective information levels, expressing a need for information on EMF health effects. Communication formats tailored to this target group should be developed and evaluated.

**Supplementary Information:**

The online version contains supplementary material available at 10.1186/s12889-026-27964-3.

## Background

In modern societies, electromagnetic fields are ubiquitous. They can be created artificially, but also occur naturally in the environment [1]. Electromagnetic fields span the entire electromagnetic spectrum, including both non-ionising and ionising radiation. For the present analysis, we focus on risk perception regarding the lower end of the electromagnetic spectrum, i.e. non-ionising fields ranging from static to radiofrequency electromagnetic fields (0 Hz-300 GHz), for reasons of conciseness hereafter called electromagnetic fields (EMFs) [1]. Even though they are classified as non-ionising radiation, EMFs can lead to some biological effects in the human body – for example, heating impacts on tissue or the stimulation of excitable cells, like muscle or nerve cells [2–4]. These effects occur above a certain field strengths [2].

To protect the population from such effects, authorities set legal limits, in Germany, for instance, based on recommendations from the International Commission on Non-Ionizing Radiation Protection (ICNIRP) and the German Commission on Radiological Protection [4–6]. The biological effects mentioned above do not lead to adverse health effects below these limits, and usually the exposure of the general population to EMFs is well below these legal limits, minimising the likelihood of adverse health effects due to exposure to EMFs [4–6]. If the limit values are taken into account, the protection of health is considered guaranteed even under chronic EMFs exposure [7].

Nevertheless, there are discussions about possible associations between EMF exposure below legal limits and various outcomes such as childhood leukaemia, brain tumours, or neurodegenerative diseases (e.g., Alzheimer’s or Parkinson’s disease), however, no consistent scientific results have been found so far [3, 8–13]. In addition, no clear conclusion concerning EMF exposure and other endpoints, such as concentration disorders, effects on female/male reproductive capacity, or non-specific symptoms such as fatigue or nervousness, can be drawn so far [11, 14–19].

However, concern about the potential effects of EMF exposure on health and lack of knowledge on this topic is relatively frequent in the general population. A representative survey from Ireland in 2023 reported that 58% of the study participants incorrectly believed that radiofrequency EMFs can damage human cells in a similar way to X-ray radiation [20]. Besides, the “Eurobarometer” survey already reported in 2010 that 46% of the EU population is very concerned about possible health risks from EMFs, and approximately 40% said that they were dissatisfied with the given information on this topic [21]. In Germany, a survey conducted in 2019 on behalf of the Federal Office for Radiation Protection reported that between 17 and 22% of the study population were very concerned about possible health effects of mobile phones, smartphones, high-voltage power lines, and mobile phone base stations [22]. In the subsequent survey in 2022, about a fifth of the population stated that they were very worried about radiation arising from smartphones, mobile phone base stations, and high-voltage power lines, while about half of the respondents said that they could not protect themselves from these types of radiation [23]. This trend continued in the latest version of the survey conducted in 2024: between 15 and 21% of the population stated they were very concerned about the radiation from smartphones, mobile phone base stations, and high-voltage power lines [24]. Concerns regarding the possible health effects of everyday technologies generating EMFs have therefore remained stable in recent years, but should not be disregarded. From a broader perspective, concerns regarding EMF and related technologies can be classified under the concept of Modern Health Worries (MHW), which are defined as “the degree to which individuals are concerned about features of modernity affecting their health” [25, 26].

If scholars, experts, science communicators, and (health) authorities aim to address these worries and adequately inform the general population about the current scientific knowledge on EMFs and health, it is important to understand the origins of concern and to examine information needs as well as ways in which information can be disseminated among the general population.

For this purpose, it is crucial to understand the risk perception, attitudes, and information level concerning potential health effects of EMF exposure among professional groups that act as central disseminators/multipliers of health-related information to members of the public [27]. School teachers and nursery school teachers are among those multipliers since they are at the forefront of providing information (including health-related information) to parents as well as to children and as a result to the general population. As a central agent of socialisation, they convey knowledge and experiences to their social networks and their environment [27, 28]. This includes the dissemination of knowledge and skills regarding environmental influences (such as EMFs) and their potential effects.

The topic of EMFs plays a very minor role in German curricula and is only very roughly discussed in physics lessons in grades 7–10, with only 12 to 16 lessons per school year [29–31]. Furthermore, a curriculum-based discussion of the potential health effects of EMFs in a school setting is highly unlikely. Accordingly, to our knowledge, school teacher training programs (with the exception of physics school teacher training) rarely address the topic of EMFs. Since, for example, primary school teachers or early childhood nursery school teachers do not need to deal with the theme of EMFs in their training or in their professional practice, the level of engagement with it is likely to be even lower in these professions.

Nevertheless, questions on this topic can arise at any time due to the high level of public concern. Therefore, they are a very important interface to the general population, also with respect to the topic of EMF exposure [27, 28, 32].

However, there is a lack of knowledge about risk perception – defined as an individual’s subjective assessment of the level of risk associated with a particular hazard [33, 34] – and the subjective levels of information regarding EMFs among school teachers and nursery school teachers, which describes how much they believe they understand with this specific topic [35, 36]. Furthermore, the extent to which these professional groups are in contact with the topic of EMF exposure in their daily working life has not yet been investigated.

As mentioned above, such knowledge about school teachers’ and nursery school teachers’ EMF perception is important, though to meet information needs in the general population and to reduce research gaps concerning the perception of potential health risks related to EMF exposure in the general public.

Against this background, this study aimed to investigate EMF risk perception, the relevance of EMFs in everyday working life, and subjective knowledge regarding EMFs among school teachers and nursery school teachers.

## Methods

We conducted a descriptive cross-sectional study across Germany using a mixed-methods design that combined a quantitative online survey with qualitative focus groups. Using a sequential explanatory design, the results of the quantitative survey – which was conducted first – were deepened by the focus groups. Both study parts were first analysed separately and combined afterwards to generate a comprehensive overall picture.

A comparable study from 2023 was carried out among German physicians, and other surveys assessing EMF-related risk perception [37–39] formed the basis for the questionnaire used in the present survey. Both the English version and the original German version are provided in the supplement (Additional file 1).

Before the fieldwork began, the questionnaire was evaluated in a pretest. This pretest aimed to determine the questionnaire’s comprehensibility and feasibility, as well as the average time required to complete it. A total of 36 people participated in the pretest, 35 of whom completed the questionnaire in full. The pretest participants were a convenience sample, meaning they were randomly invited and readily available to us. Based on the results, minor adjustments were made, such as adding a few multiple-choice options to some items. The clarity of the questions, as well as the planned response time of approximately 15 min, was confirmed.

The study was approved by the Ethics Committee at the Medical Faculty of LMU Munich (project number 24–0204). All participants gave informed consent to take part in the study. The first page of the online survey contained all relevant information concerning participation in the study (e.g., data protection measures). Participants had to confirm that they have read and understood these instructions and consent to participating in the survey by ticking a digital “I agree” checkbox before starting the survey. For the online focus groups, a similar approach was used: Before the start of the discussion, the information about the study was presented and displayed. Next, participants were required to tick a checkbox to confirm that they have read and understood these instructions and consent to participating in the group discussions.

### Study population

The source population underlying our study population consisted of school teachers and nursery school teachers from general education schools, vocational schools, day nurseries, kindergartens, and other educational institutions that work with children and adolescents. Applying a convenience sampling approach, study invitations were distributed from June to October 2024 via social media, advertising in newspapers, and by contacting relevant federal and state associations with the request to spread information about the study. Furthermore, we sent 2400 postal invitations to a quasi-randomised selection of school facilities and day nurseries to ensure that participants were recruited from every federal state. Participants who finished the online survey did receive compensation in the form of an online shopping voucher (25€ for completion of the survey).

### Outcomes

There were three outcomes of interest: risk perception regarding EMFs, subjective information level on EMFs, and the relevance of this topic in participants’ everyday work.

Risk perception concerning potential health impacts caused by EMFs was measured by participants’ degree of agreement with two statements. First, participants were asked whether they believed that “There are individuals who develop adverse health effects from electromagnetic fields below legal limits” (hereafter: EMF health effects below legal limits). This item should address the physical aspects and thus the direct effects of EMF exposure on health when the legal limits are observed. The second statement was “Adverse health effects from electromagnetic fields can also have non-physical causes” (hereafter: EMF health effects can also have non-physical causes). In contrast to the first item, the second item should cover the psychosomatic aspects. Participants were asked to indicate their agreement to each statement on a 5-point-Likert scale ranging from 0 = “I do not agree” to 4 = “I agree”. For further analyses, these two variables were dichotomised (agreement vs. no agreement), with the middle category being defined as “no agreement” in order not to overestimate the degree of agreement. To verify the robustness of our results, a sensitivity analysis was also carried out for the variable “EMF health effects below legal limits”, for which the middle category was considered agreement.

All participants who agreed on the statement regarding EMF health effects below legal limits were asked two additional questions: “In your opinion, which adverse health effects can be caused by electromagnetic fields?” (hereafter: health effects) and “In your opinion, which sources produce electromagnetic fields that can cause adverse health effects?” (hereafter: EMF sources). Participants could choose one or more of these answer options for health effects: headaches, sleeping disorders, nervousness/restlessness, difficulties concentrating, ADHD/behavioural problems, vertigo, tinnitus/hearing disorders, vision disorders, fatigue, cardiac arrhythmias, cancer, Alzheimer’s disease. For EMF sources, participants could pick one more of these answer options: mobile phones, mobile phone base stations, cordless landline phones, radio/television, WiFi/Bluetooth/computer, microwave, induction cooker, power lines, digital boards/whiteboards. In addition, both questions offered the option to add further answers using free text.

To assess the relevance of EMFs in everyday working life, participants were asked whether health effects of EMFs have ever been discussed at their work (yes/no) and, if so, how often this has happened during the last 12 months (answer options: 0 times, 1–4 times, 5–9 times, 10–49 times, 50–99 times, ≥ 100 times) and on what occasion (answer options “as part of lessons”, “at parents’ evenings”, “during discussions with parents/guardians”, “during discussions with colleagues”, “when children/young people raise the topic” or “other”). To improve the clarity of the results presented here, the frequency of occurrence of the topic of health effects of EMF was categorised as never (0 times), rarely (1–4 times), occasionally (5–9 times) and frequently (10 times or more).

Participants’ subjective information level was measured by asking, “How well do you feel informed about potential health effects of electromagnetic fields?”. Participants could answer on a 5-point-Likert-Scale from 0 = “poorly informed” to 4 = “well informed”. For further analysis, answers were dichotomised (poorly informed vs. well informed), with the middle category being classified as “well informed” because we specifically were interested in identifying the proportion of school teachers and nursery school teachers who considered themselves as poorly informed. Furthermore, a sensitivity analysis was conducted for this item, in which the middle answer option for this item was defined as poorly informed.

This statement was followed by the question “Have you received any new information about the health effects of EMFs during the past 12 months?”. Here, there were three answer options: “Yes, I searched actively for information” (information seeking), “Yes, I received information randomly/passively (e.g., through radio broadcasts or articles in magazines)” (information scanning), or “No, I neither searched for information actively nor did I receive information by chance”. It was possible to choose both yes answers at the same time. In case of at least one positive answer, another question asked the participants which sources had provided them with information about EMFs (hereafter: source of information). Here, too – in addition to the option of a free text answer – various possible answer options were provided: public broadcasting, private broadcasting, regional tabloid newspapers, nationwide tabloid newspapers, regional quality newspapers, nationwide quality newspapers, social media posts, comments of internet users, alternative media (EMF critical), websites of public organisations, articles in scientific journals.

### Correction variables

Furthermore, we collected information on different demographic variables: gender (female, male, diverse), age (< 20 years, 20–29 years, 30–39 years, 40–49 years, 50–59 years, ≥ 60 years), federal state (all 16 German states), type of municipality in which participants’ institution is located (large town: ≥ 100,000 inhabitants; medium town: < 100,000 & ≥ 20,000; small town: < 20,000 & ≥ 10,000; village: < 10,000 & ≥ 5,000; rural municipality: < 5,000), and sponsorship of their institution (public, private, or other) [40]. In addition, school teachers were asked about the type of school they work at, while nursery school teachers were asked about the age of the children they mainly work with (< 3 years, 3–6 years, 7–10 years, 11–14 years, ≥ 15 years).

### Focus groups

After finishing the quantitative survey, the participants were asked if they wanted to take part in additional focus groups. From everyone interested, randomised samples were drawn, consisting of school teachers and nursery school teachers from every federal state and different kinds of work facilities.

The guideline for the focus groups was developed based on the previously obtained results of the quantitative online survey and the scientific literature. Based on the current state of research, we first deductively derived categories that would yield relevant results from a communication studies perspective: “professional and private media use”, “media health literacy”, and “professional and private technology acceptance”. These three categories were then supplemented by data on participants’ EMF risk perception, relevance of EMFs in everyday working life, and subjective information level that were collected in the online survey.

Before data collection, the interview guide was also pretested. Participants included a preschool teacher, a school teacher from a private school, and a school teacher from a public high school. All questions in the guide were asked, and participants were asked to provide feedback on the questions (order, wording, and any potentially missing aspects). No issues were identified, so no adjustments to the interview guide were necessary. The pretests lasted between 18 and 31 min. Participants received an expense allowance of 50 euros for taking part in the pretest.

The discussions took place between November and December 2024. Two moderators led these online discussions (via video conferencing), in which four school teachers or nursery school teachers located all over Germany participated each. The duration of the focus groups varied between 47 and 100 min. To ensure accurate data capture, the interviews were video recorded. They were then transcribed verbatim using the automated, GDPR-compliant transcription tool f4x [41]. All statements were completely anonymised (removing all personal data, including school names and other identifying information) to ensure data protection. Transcripts were coded qualitatively [42] using MAXQDA version 2024 [43], with deductive categories supplemented inductively. This approach ensures accurate representation while minimising researchers’ biases [44]. Two authors conducted the coding and then actively discussed their respective coding results to achieve agreement. The coded transcripts were selectively retrieved and organised to enable systematic comparison, through which we identified repeating patterns [45]. All participants who took part in the focus groups did receive compensation in the form of another online shopping voucher (100€ for participating in the focus groups).

### Quantitative survey: statistical analysis

The socio-demographic data of the source population and the study population are presented descriptively with absolute and relative frequencies. “Health effects” and “EMF sources” are reported descriptively, too.

For the outcome variables, we calculated prevalence estimates with 95% confidence intervals (CI) for proportions. Furthermore, their prevalence estimates were corrected based on the assumption that the study population can be divided into different groups, with each participant assigned to exactly one group. Every group was defined by the correction variables professional group, gender, age, and federal state (additional, school type for school teachers). The correction was implemented using Multilevel Regression and Poststratification (MRP), which consisted of two steps: First, the prevalence in each different group was estimated using Bayesian multilevel models [46, 47]. The models contained the above-mentioned correction variables as predictors/independent variables. In the second step, an overall estimate was calculated from the individual group estimates by weighting. This required information on the distribution of every population group in the source population [46, 47]. Data on the distribution of the source population came from the Federal Statistical Office from the Statistical Report on General Education Schools (school year 2022/2023) [48] and from the Statistical Report on Vocational Schools and Schools of the Health Sector (school year 2022/2023) [49]. Estimates were summarised by median (point estimate) and 95% posterior interval (interval estimate). The models of all outcomes were adjusted for age, gender, federal state of working place, and, for school teachers, additionally for the type of school they work in.

Besides, a latent class analysis (LCA) was used to identify different types of EMF risk perception. LCA is a statistical method that creates clusters based on answer patterns of several categorical variables [50]. In our case, these patterns arose from the answers to the variables “EMF health effects below legal limits”, “EMF health effects can also have non-physical causes”, “health effects”, and “EMF sources”.

We selected “EMF health effects below legal limits” and “EMF health effects can also have non-physical causes” as we considered them as key indicators for EMF-related risk perception [38, 39]. Furthermore, earlier studies showed that individuals can differ in their appraisal of different sources of EMF as well as diseases/symptoms attributed to EMFs. Against this background, we selected these two as additional indicator variables [38, 39, 51].

Every one of these clusters was equal to one category of EMF risk perception. The model was calculated for 2 to 7 classes, and the best model was selected from the available models. Models that were not identified, i.e., whose global maximum likelihood solution could not be found, were excluded. Based on statistical criteria (Akaike Information Criterion (AIC), Bayesian Information Criterion (BIC)) and due to interpretability, a 5-category-solution was chosen. For every participant, a list of probabilities (based on their survey responses) with which a person is part of a certain risk perception category was calculated. This assumes that every single participant can be assigned to exactly one risk perception category, so the sum of the probabilities is 1. The calculation of the category assignment was carried out 20 times per participant. The mean and 5% and 95% percentiles of these 20 frequencies, means, and standard deviations are reported. Additionally, the average posterior classification probabilities and the entropy were calculated as measures for the quality of class separation in the LCA.

Furthermore, sensitivity analyses were conducted. Especially at the beginning of the study, some questionnaires were apparently completed by bots – computer programmes that operate automatically on the internet without human intervention [52]. Therefore, some measures were implemented to exclude existing bot-questionnaires and to prevent new bots from participating. Exclusion criteria were, for example, the language of answers to free-text questions or filling out the questionnaire in under 5 min. Moreover, we added a CAPTCHA before the start of the survey and created a new link (URL) to the same survey that was used for recruitment via postal invitation. Accordingly, participants who had received the new link to the survey via mail were very likely not bots. Sensitivity analyses based on the two study links (URLs) were conducted for the outcome variables EMF risk perception, relevance in everyday work, and subjective level of information to verify the robustness of the results (entire study population vs. study population invited via postal invitation).

Analysis of the quantitative data was conducted in R [53] including the usage of the rstanarm package [54] and Stan for the calculation of Bayesian models [55]. For each model, we had 4000 samples available. The quality of the sampling process was ensured by checking the diagnostic criteria [56]. For the calculation of the latent class analysis, the R package poLCA was used [57].

## Results

In total, 1400 school teachers, nursery school teachers, and other educational personnel working with children and youth participated in the survey (Table 1). Both in the source population, the population from which the participants came, and the study population, the number of school teachers was higher than the number of nursery school teachers.

**Table 1:** Distribution o s by population

Variable	Study population					Source population			
	Total	School teachers		Nursery school teachers		School teachers		Nursery school teachers	
Total	1 400*	1 053 (75.2%)		329 (23.5%)		925 264		744 683	
Gender									
Female	931 (66.5%)	666 (63.2%)		256 (77.8%)		655,922 (70.9%)		684,350 (91.9%)	
Male	459 (32.8%)	380 (36.1%)		70 (21.3%)		269,342 (29.1%)		6,333 (8.1%)	
Diverse	8 (0.6%)	5 (0.5%)		3 (0.9%)		-		-	
Age group									
< 20 years	8 (0.6%)	4 (0.4%)		4 (1.2%)		87,240** (9.4%)		197,842** (26.6%)	
20–29 years	175 (12.5%)	101 (9.6%)		74 (22.5%)					
30–39 years	441 (31.5%)	333 (31.6%)		103 (31.3%)		259,959 (28.1%)		183,231 (24.6%)	
40–49 years	391 (27.9%)	304 (28.9%)		81 (24.6%)		244,105 (26.4%)		165,711 (22.3%)	
50–59 years	288 (20.6%)	230 (21.8%)		54 (16.4%)		234,129 (25.3%)		143,565 (19.3%)	
≥ 60 years	97 (6.9%)	81 (7.7%)		13 (4.0%)		99,831 (10.8%)		54,334 (7.3%)	
Sponsorship									
Public	1163 (83.1%)	929 (88.2%)		222 67.5%)		-		-	
Private	237 (16.9%)	124 (11.8%)		107 (32.5%)		-		-	
School type									
Elementary School	-	130 (12.3%)		-		250,431 (27.1%)		-	
Secondary/Middle School	-	84 (8.0%)		-		31,495 (3.4%)		-	
Secondary Modern School	-	223 (21.2%)		-		63,399 (6.9%)		-	
Grammar School	-	327 (31.1%)		-		206,788 (22.3%)		-	
Comprehensive School	-	53 (5.0%)		-		110,433 (11.9%)		-	
School type with multiple educational exits	-	20 (1.9%)		-		49,179 (5.3%)		-	
Free Waldorf School	-	30 (2.8%)		-		9,084 (1.0%)		-	
Special school	-	50 (4.7%)		-		80,932 (8.7%)		-	
Further Education Colleges	-	110 (10.4%)		-		123,523 (13.4%)		-	
Others	-	3 (0.3%)		-		-		-	
Age of the children to be cared for									
< 3 years	-	-		140 (42.6%)		-		-	
3–6 years	-	-		216 (65.7%)		-		-	
7–10 years	-	-		44 (13.4%)		-		-	
11–14 years	-	-		23 (7.0%)		-		-	
≥ 15 years	-	-		14 (4.3%)		-		-	
Type of municipality									
< 5.000	170 (12.1%)	93 (8.8%)		74 (22.5%)		-		-	
5.000–10.000	210 (15.0%)	147 (14.0%)		62 (18.8%)		-		-	
10.000–20.000	303 (21.6%)	229 (21.7%)		69 (21.0%)		-		-	
20.000–100.000	353 (25.2%)	297 (28.2%)		52 (15.8%)		-		-	
> 100.000 inhabitants	362 (25.9%)	286 (27.2%)		71 (21.6%)		-		-	

*The difference between the total study population and the total number of participating school teachers and nursery school teachers corresponded to nursery school teachers who were included in the study but who could not be assigned to the professional group of school teachers or nursery school teachers (*N* = 18)

**All under-30s of the source population were grouped together

Overall, the comparison of the distribution of socio-demographic variables between the source and study populations showed no notable differences. Only the proportion of men among the nursery school teachers in the study population (21.3%) was slightly higher than that of the source population (8.1%). Most of the participants were between 30 and 49 years old (school teachers: 60.5%; nursery school teachers: 55.9%). The sponsorship of the participants’ institution was mostly public. While the distribution of types of municipalities among nursery school teachers was comparatively balanced, school teachers from municipalities with fewer than 10,000 inhabitants were less frequently represented in the study population.

The study population of the qualitative focus groups consisted of 29 participants (17 school teachers and 12 nursery school teachers). While 17 men participated in the discussions, women were somewhat less represented with 11 participants. In addition, one gender diverse person participated. Most participants were aged between 28 and 39 years, and the sponsorship of the workplace was, in most cases, public.

With a proportion of 27.1%, most participants choose the Likert-Scale value 0 and therefor did not agree with the statement regarding “EMF health effects below legal limits” (see supplement table S1). The middle category of the Likert-Scale was the second most frequently chosen option for this item (23.2%) − 16.2% of the participants indicated full agreement.

**Fig. 1:**
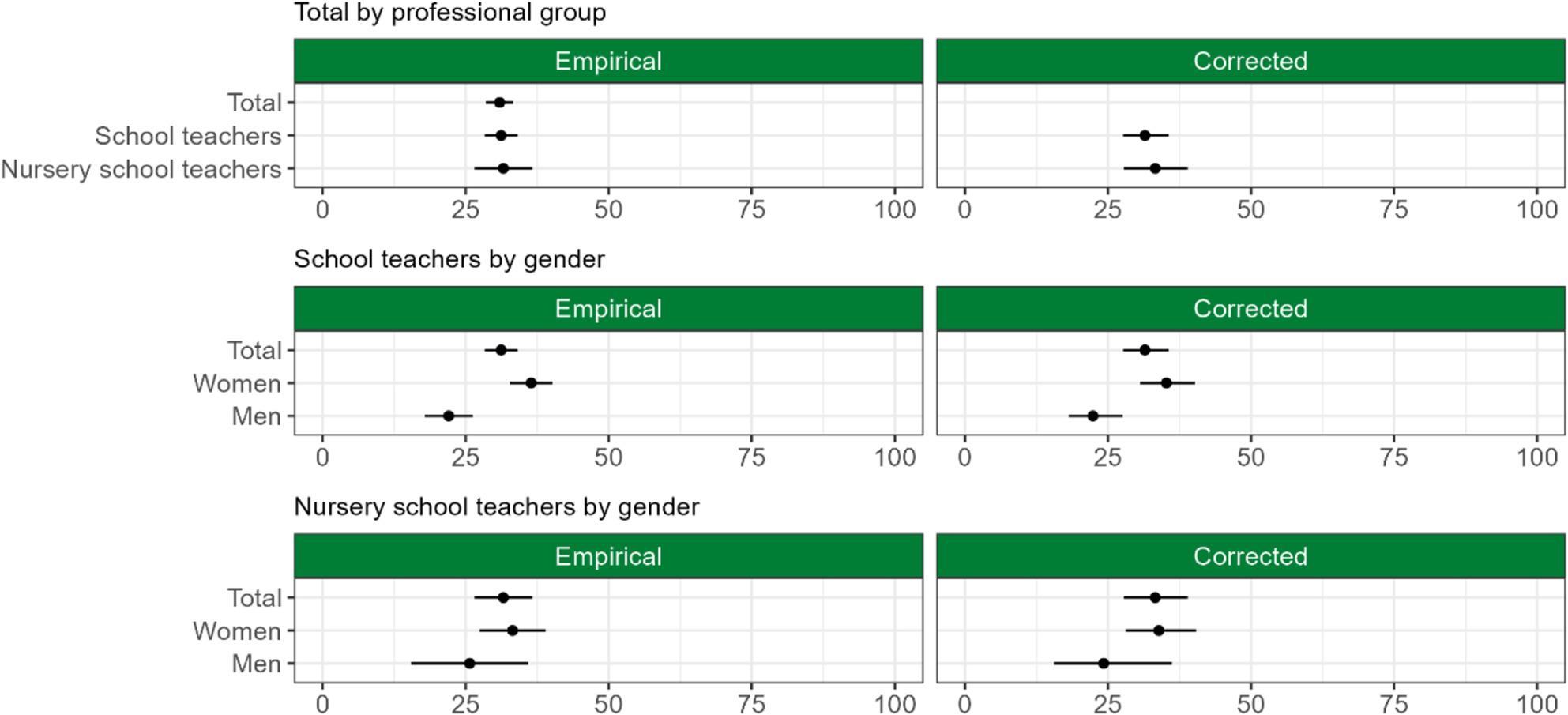
EMF health effects below legal limits (Agreement in %). Proportion of participants who agreed with the statement regarding EMF health effects below legal limits; Total by professional group and by gender for school teachers and nursery school teachers: empirical in the study population and corrected for non-response using MRP for source population

The proportion of participants who partly or fully agreed with the statement regarding “EMF health effects below legal limits” was similar across both professional groups, with 31.2% (95%-CI: 28.4%–34.1%) among school teachers and 31.6% (95%-CI: 26.5%–36.6%) among nursery school teachers (Fig. 1).

There was no difference between these empirical estimates and the corrected prevalence estimates of 31.5% (95%-CI: 27.7%–35.6%) for school teachers and 33.3% (95%-CI: 27.7%–38.9%) for nursery school teachers. Female school teachers agreed with the statement regarding EMF health effects below legal limits slightly more often (35.2%, 95%-CI: 30.6%–40.2%) than male school teachers (22.4%, 95%-CI: 18.1%–27.5%) (Fig. 1).

This observation was somewhat less pronounced among nursery school teachers, where 33.9% (95%-CI: 28.1%–40.4%) of the women and 24.2% (95%-CI: 15.5%–36.1%) of the men agreed with the statement regarding EMF health effects below legal limits (Fig. 1).

In the sensitivity analysis that interpreted the middle category as agreement, over half of the school teachers (51.7%) and nursery school teachers (62.3%) did not rule out health effects from EMF below legal limits with considerable gender differences (see table S 2 in supplement).

Looking at the descriptive values of the item “EMF health effects can also have non-physical causes”, it was found that almost equal proportions of participants (21.1%, 23.6% and 24.3% respectively) opted for the two extreme response options and for the middle category (see supplement table S 1).

**Fig. 2:**
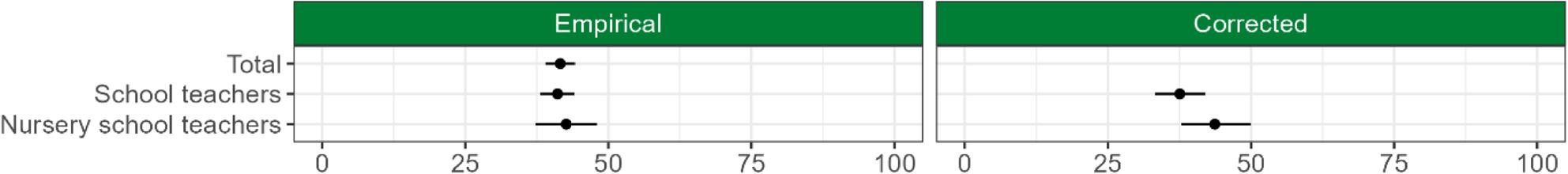
EMF health effects can also have non-physical causes (Agreement in %). Proportion of participants who agreed with the statement weather EMF health effects can also have non-physical causes; Total and by professional group: empirical for study population and corrected for non-response using MRP for source population

There were only small differences in the answer pattern of participants of both professional groups regarding the statement whether EMF health effects can also have non-physical causes. Here, an (corrected) estimated 37.6% (95%-CI: 33.2%–42.0%) of school teachers and 43.7% (95%-CI: 37.8%–50.0%) of nursery school teachers agreed (Fig. 2).

The calculation of the Pearson correlation between both items regarding EMF risk perception yielded a coefficient of 0.42 at a p-value of < 2.2e-16, thus indicating a moderately strong correlation.

**Fig. 3:**
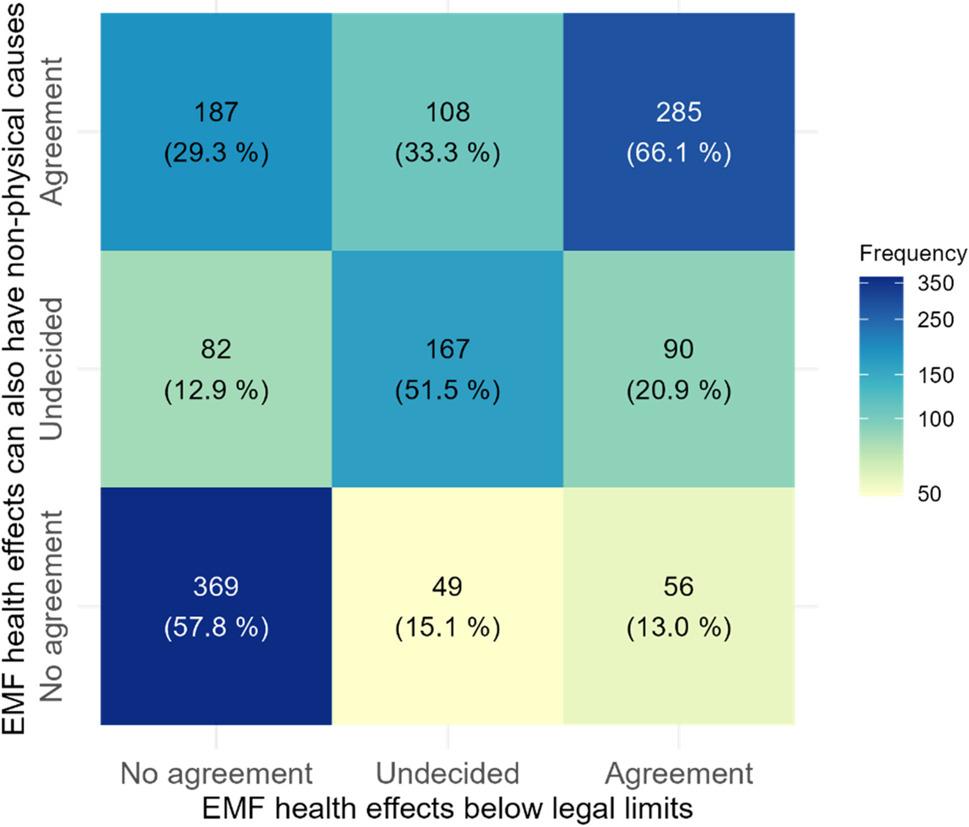
Heatmap: Absolute frequencies of combined agreement with both statements regarding the EMF risk perception. "Disagree" and "Agree" each represent the two most extreme response options on the 5-point Likert scale, "Undecided" corresponds to the middle response option; participants with missing values for at least one of the two statements were excluded; relative frequencies refer to the columns; *N*=1393

The heatmap visualises that the most frequent response combination, with an absolute frequency of 369, was disagreement with the statement regarding EMF health effects below legal limits and disagreement with the statement regarding the item “EMF health effects can also have non-physical causes” (Fig. 3).

Agreement with both statements was the second most frequent combination. However, all other response combinations also occurred at least 49 times, with the combination of disagreement with the statement regarding EMF health effects below legal limits and agreement with the statement regarding non-physical causes of health effects (187) and the combination of “undecided” and “undecided” (167) being somewhat more prominent.

**Fig. 4:**
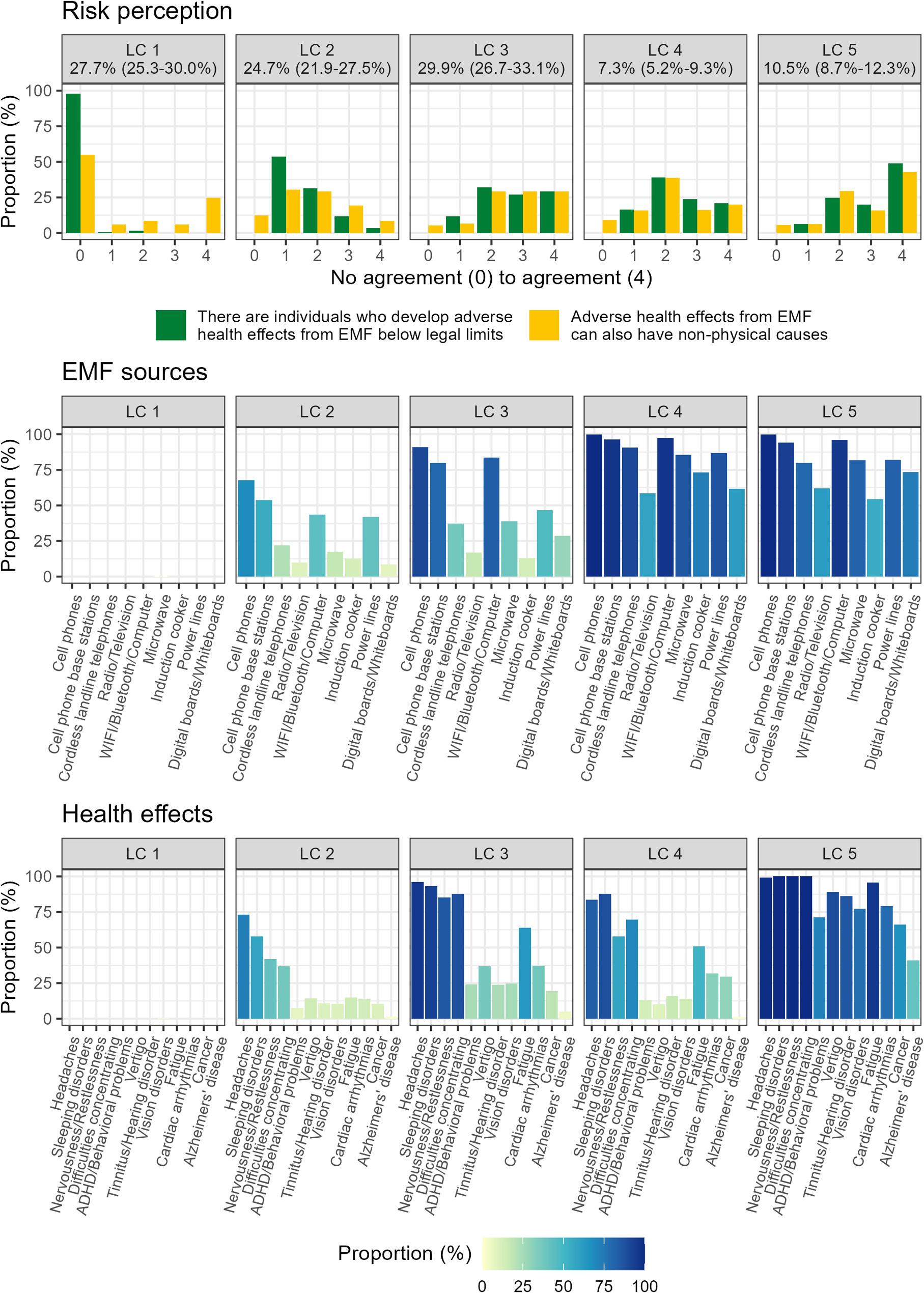
Latent class analysis of EMF risk perception

In the LCA, five classes were identified: Type 1 (LC 1): Individuals with low risk perception; Type 2 (LC 2): Individuals with rather low risk perception regarding selected EMF sources and selected health problems; Type 3 (LC 3): Individuals with medium to rather high risk perception regarding selected EMF sources and selected health problems; Type 4 (LC 4): Individuals with medium to rather high risk perception regarding all EMF sources surveyed and selected health problems; Type 5 (LC 5): Individuals with high risk perception regarding all EMF sources and all health problems (Fig. 4).

The prevalence of LC 1 was estimated at 27.7% (95%-CI: 25.3%–30.0%) of the participants. In this class, the values of the variables of health effects and EMF sources were 0%, as these questions were not shown to participants who did not agree to the statement of EMF health effects below legal limits. LC 2 had a prevalence of 24.7% (95%-CI: 21.9%–27.5%). LC 3 had the highest prevalence with 29.9% (95%-CI: 26.7%–33.1%), while LC 4 had the lowest prevalence with 7.3% (95%-CI: 5.2%–9.3%). The prevalence of participants with high risk perception (LC 5) was 10.5% (95%-CI: 8.7%–12.3%). The model parameters shown in Fig. 4 are also listed in table S 3 in the supplement.

Regarding the five different classes, there were also differences in socio-demographic variables. The proportion of nursery school teachers in LC 5 was, for example, considerably higher than in the other four classes. Conversely, the proportion of school teachers in LC 5 was lowest. Regarding gender, the proportion of women (79.4%; 95%-CI: 77.5%–81.7%) in the class with the highest risk perception (LC 5) was four times higher than the proportion of men in the corresponding class (20.6%; 95%-CI: 18.3%–22.5%). In contrast to every other class, participants of LC 1 stated with a proportion of 42.3% (95%-CI: 42.2%–42.5%) a clearly lower subjective information level. A comprehensive overview can be found in table S 4 in the supplement. The diagonal elements of the Average Posterior Classification Probability matrix ranged from 0.85 (LC 5) to 0.99 (LC 1), indicating high assignment accuracy and distinctiveness of the identified classes (see supplement table S 5). The observed entropy value of 0.89 indicated a good separation between the latent classes and a high degree of classification certainty.

**Fig. 5:**
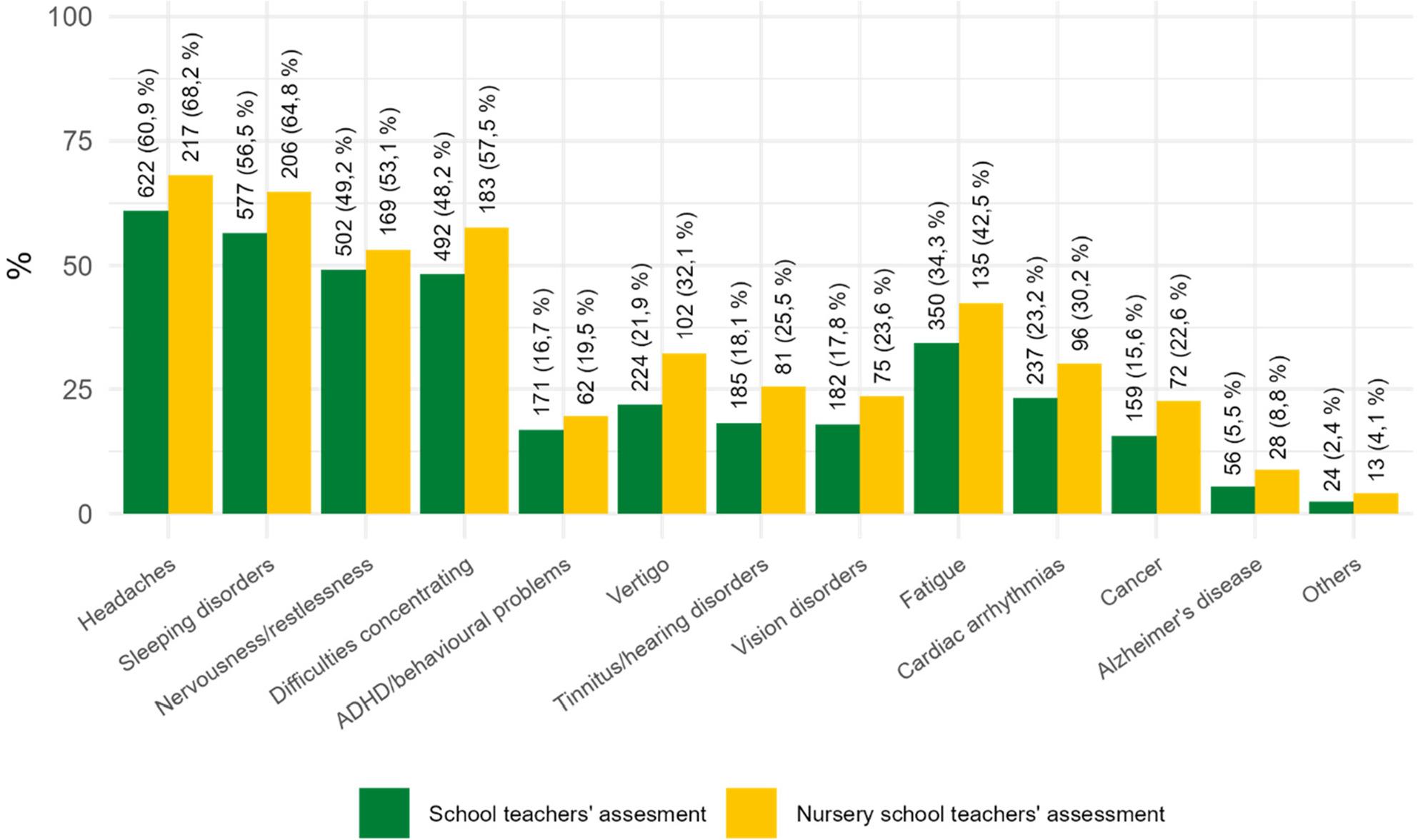
Assessment by all participants that corresponding health symptoms can be caused by EMF. Online survey – Absolute and relative frequencies of all participants' assessment that corresponding health complaints can be caused by EMF; by professional; multiple-choice answers; N school teachers = 716, N nursery school teachers = 247

Participants who at least partly agreed with the statement regarding EMF health effects below legal limits answered the question about health effects due to EMF exposure from their perspective mainly with non-specific symptoms like headaches (school teachers: 60.9%; nursery school teachers: 68.2%), sleeping disorders (school teachers: 56.5%; nursery school teachers: 64.8%), nervousness/restlessness (school teachers: 49.2%; nursery school teachers: 53.1%), and difficulties concentrating (school teachers: 48.2%; nursery school teachers: 57.5%) (Fig. 5).

The frequency with which EMFs were associated with pathological diseases such as cardiac arrhythmias (school teachers: 23.2%; nursery school teachers: 30.2%), cancer (school teachers: 15.6%; nursery school teachers: 22.6%), and Alzheimer’s disease (school teachers: 5.5%; nursery school teachers: 8.8%), was much lower.

In the group discussions, both school teachers and nursery school teachers reported specific health complaints that they might associate with EMFs. These included headaches, sleep disturbances, and difficulty falling and staying asleep (e.g., “Headaches, of course. And dizziness can occur. […] It’s hard to concentrate” – female nursery school teacher, 44 years). School teachers often expressed uncertainty about whether and to what extent these symptoms were actually attributable to EMFs.

The (partially) EMF-concerned participants of both professional groups mostly named mobile phones (school teachers: 58.5%; nursery school teachers: 68.2%), mobile phone base stations (school teachers: 51.9%; nursery school teachers: 57.9%) and WiFi/Bluetooth/computers (school teachers: 50.8%; nursery school teachers: 57.9%) as relevant EMF sources.

The focus groups largely confirmed these results. Most respondents in the discussions – both school teachers and nursery school teachers – did not expect health issues due to EMF exposition. However, uncertainties regarding long-term effects – especially on children – were mentioned. In addition, participants discussed whether possible arising symptoms like headaches or sleeping disorders are actually due to EMFs or whether they could have been triggered by other factors. One school teacher, for example, reacted to this issue by saying “Well, I have a WiFi socket up here, and it only annoys me because it blinks, that’s it [laughs]. Otherwise, I think it’s completely unproblematic”.

**Fig. 6:**
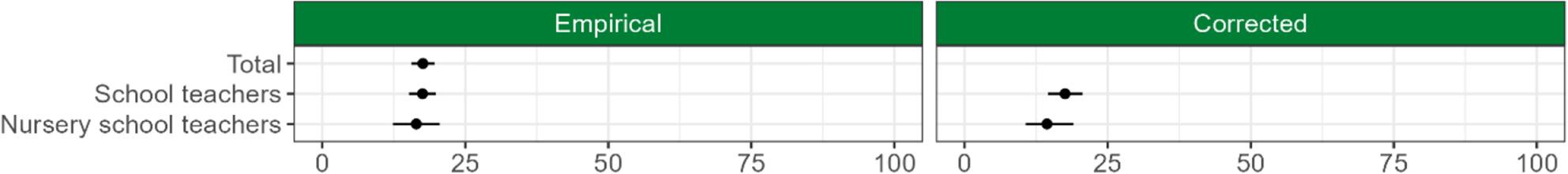
Relevance of EMF in everyday working life (Proportion of "ever relevant" in %)

The reported relevance of the health effects of EMFs in everyday working life was generally low. Only 17.6% (95%-CI: 14.6%–20.6%) of the participating school teachers and 14.4% (95%-CI: 10.7%–19.0%) (corrected values) of the nursery school teachers stated that this topic ever came up at work (Fig. 6).

Participants who indicated that this topic had ever arisen during work predominantly stated that it had occurred rarely (1–4 times) within the last 12 months (school teachers: 56.6%; nursery school teachers: 50.9%) – mainly in conversations with colleagues (school teachers: 74.9%; nursery school teachers: 60.0%). School teachers otherwise reported that this topic arises in the context of lessons (29.8%), while nursery school teachers second most frequently cited parent evenings (32.7%). The proportion of school teachers who frequently encountered this topic in their daily work was 10.2%, while the proportion of nursery school teachers who frequently dealt with the topic was 15.6%.

Except for isolated intensive discussions on the topic of EMFs and possible health effects – which were mostly triggered by colleagues – the group discussions also pointed towards little relevance of the topic to everyday working life among school teachers and nursery school teachers.

There were isolated instances of intense conflict among school teachers, mostly triggered by colleagues who, for example, pointed out issues like WiFi or protective clothing such as radiation-shielding underwear, as one female, 40 years old school teacher reported: “I have a colleague who wears radiation protection underwear. […] And he always switched off the Wi-Fi in every room he was in. This was, of course, very annoying for his colleagues”. Parental perspectives also played a role in the daily school life of school teachers, with, for example, a 26-year-old school teacher reporting on a mother who attributed her son’s learning difficulties to his seat being close to the WiFi router and demanded a change of seat – without this having any effect on improved school performance. Nursery school teachers described hardly any direct points of contact in their daily work, but they occasionally came across the topic in conversations among colleagues. A 28-year-old nursery school teacher emphasised: “I only heard about it privately, but it wasn’t actually discussed in the daycare center”. Some described how the topic was “flooded into the feed” on social media or through algorithms (nursery school teacher, male, 38 years), but this hardly led to any in-depth discussion. Although isolated situations could be identified in which the topic of health and EMFs was present, overall, it remained a marginal aspect.

**Fig. 7:**
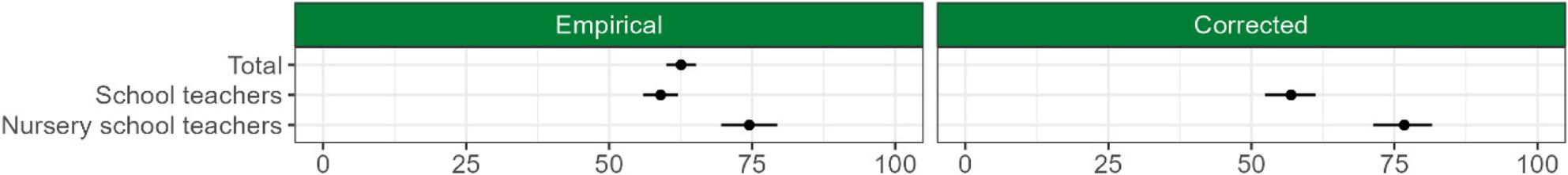
Poor subjective knowledge regarding EMF (Agreement in %). Proportion of school teachers and nursery school teachers with poor subjective knowledge regarding EMF. Total and by professional group: empirical for study population and corrected for non-response using MRP for source population

56.9% (95%-CI: 52.4%–61.3%) of the school teachers and 76.7% (95%-CI: 71.3%–81.6%) (corrected values) of the nursery school teachers indicated they felt poorly informed about possible health effects of EMFs (Fig. 7 and table S 6 in the supplement).

In the sensitivity analysis recoding, the mean response option for this variable to “poorly informed”, the proportions of subjectively poorly informed participants were 75.5% (95%-CI: 72.8%–78.2%) for school teachers and 90.8% (95%-CI: 87.6%–94.1%) for nursery school teachers (supplement table S 7).

Furthermore, most study participants (school teachers: 80.1%; nursery school teachers: 76.9%) stated that they had not received any information on the health effects of EMFs during the last 12 months. Nursery school teachers tended to receive information on this subject somewhat more frequently – both through active searches (11.0%) and through passive encountering of information (15.6%). However, there was no meaningful difference to the school teachers (information seeking: 7.9%; information scanning: 13.5%).

**Fig. 8:**
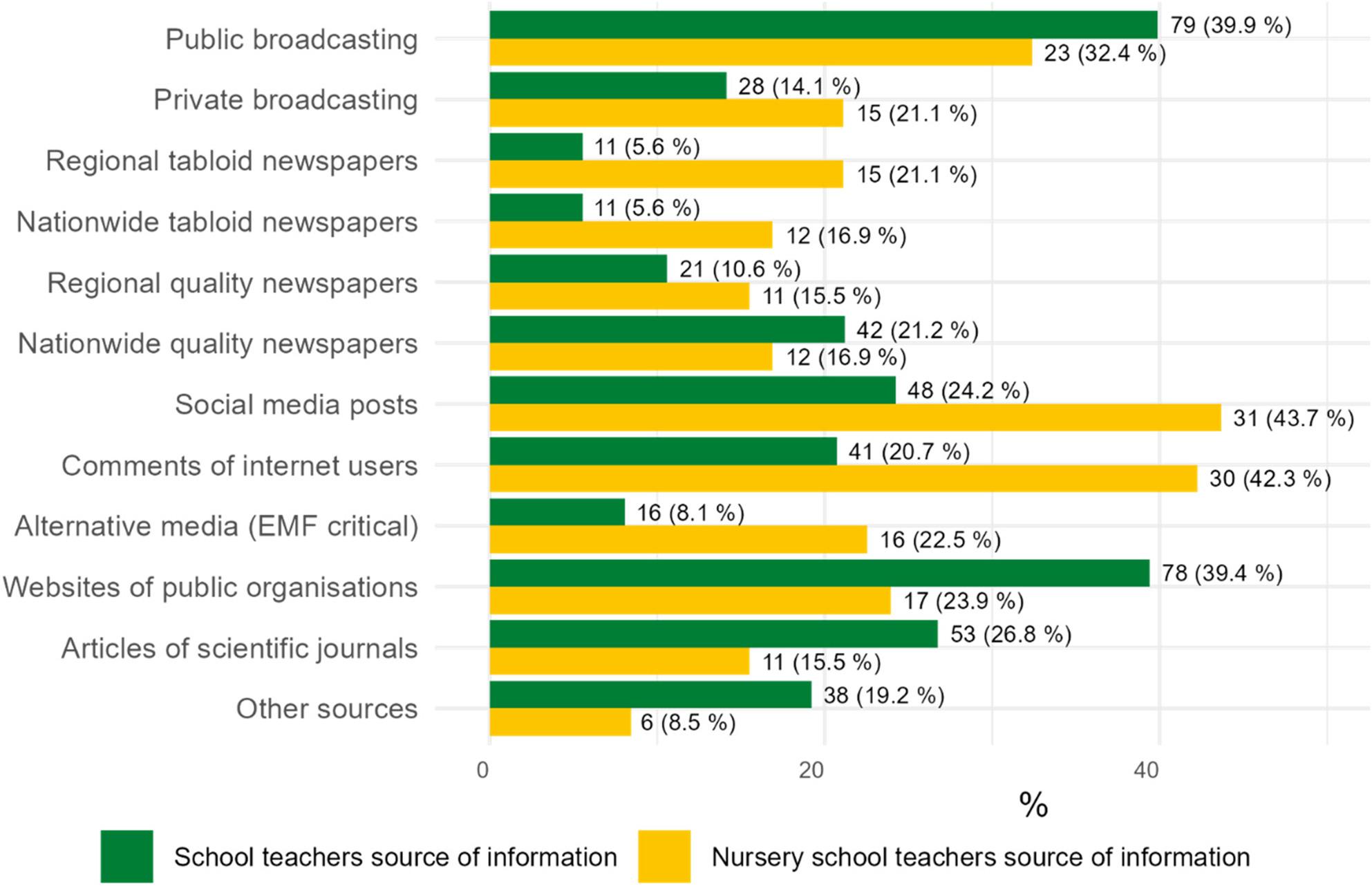
Source of information. Absolute and relative frequencies of school teachers’ and nursery school teachers’ sources of information on EMF and health in the last 12 month; by professional group; multiple answers possible

Overall, the kind of source from which the participants had obtained information regarding possible health effects of EMFs in the last 12 months differed between the two professional groups. While school teachers most frequently reported obtaining EMF-related information from public broadcasting (39.9%), websites of public organisations (39.4%), and articles in academic journals (26.8%), nursery school teachers stated social media posts (43.7%), comments from internet users (42.3%), and public broadcasting (32.4%) as their primary sources of information regarding EMFs and health (Fig. 8).

Furthermore, a higher percentage of nursery school teachers (22.5%) reported using alternative media than school teachers (8.1%).

Regarding their need for information about EMFs, school teachers and nursery school teachers mentioned various aspects. In addition to information about possible physical effects – for example, headaches/migraines or infertility – they particularly desired insights into a potential cancer risk associated with EMFs. Several participants emphasised that the “long-term consequences” are particularly difficult to assess and therefore education is of high relevance. Psychological effects were also discussed, such as sleep disturbances, difficulty concentrating, and depressive moods, which have been linked to EMFs. Furthermore, many respondents expressed a desire for basic information on the physical aspects of EMFs. This includes explanations of frequencies, types of radiation, and current limits, as well as background information on everyday sources such as WiFi routers and mobile phones. A physics school teacher summed it up: “You always have to consider what power output is relevant. […] With WiFi frequencies, we can rest easy; they’re 2.5 or 5 gigahertz. These are unproblematic frequencies” (school teacher, male, 54 years).

In the group discussions, both school teachers and nursery school teachers expressed a clear need for scientifically sound and practical information on EMFs. Both professional groups also indicated a desire for more research and transparent education, with school teachers focusing more on scientific studies and practical application, while nursery school teachers demanded specific information on risks for children, for example “I would be interested to know if children are affected differently by EMFs, because they might be more sensitive” (nursery school teacher, diverse, 27 years).

Besides the content, the format of knowledge transfer was addressed by the participants. The central concern of both groups was the comprehensibility and applicability of information material. Practical formats that can be easily integrated into lessons were also desired. In this regard, structured handouts, information sheets, and digital media formats, such as short explanatory videos, were discussed. School teachers and nursery school teachers in the discussions also mentioned social media and YouTube as potentially useful and effective information channels. In general, according to the participants, information should be accessible, understandable, and presented in a variety of media, with the scientific accuracy of the content always forming a basis. Due to the complexity of the topic of EMFs and health, participants identified that tension arose between providing accurate information about the state of research and the desire for simple information.

The sensitivity analysis regarding our populations revealed largely no relevant differences in the results between the participants invited by mail and the participants recruited online. There were only marginal differences in the prevalence estimates for subjective information level (corrected prevalence estimator of the whole study population: school teachers: 56.9% (95%-CI: 52.4%–61.3%); nursery school teachers: 76.7% (95%-CI: 71.3%–81.6%); corrected prevalence estimator of the population that was invited via mail: School teachers: 68.0% (95%-CI: 60.3%–75.0%); Nursery school teachers: 81.2% (95%-CI: 72.3%–88.2%) (see supplement table S 8).

## Discussion

The present study aimed to investigate the EMF risk perception and knowledge regarding EMFs, as well as the relevance of this topic in everyday working life among school teachers and nursery school teachers. Approximately one-third of all participating school teachers and nursery school teachers agreed to the statement that EMF exposure below legal limits can cause adverse health effects. In most cases, nonspecific symptoms like headache, sleeping disorders, and difficulties concentrating were mentioned as possible effects caused by EMFs.

Our results show that nursery school teachers exhibit a higher EMF risk perception regarding the item on non-physical causes and simultaneously use alternative, EMF-critical media more frequently (22.5%) to obtain information than school teachers (8.1%). Several explanations can be discussed for this finding. First, differences in formal educational attainment are conceivable. Unlike teachers, nursery school teachers are not required to have an academic degree in Germany, which is why their educational attainment varies compared to teachers. A lower level of education could be associated with increased use of alternative media, in which EMFs are more frequently portrayed as a health risk. Studies suggest that the use of alternative media or belief in conspiracy theories increases with decreasing levels of education [58, 59]. Second, nursery school teachers indicated a lower subjective information level of EMF compared to school teachers. This could contribute to a higher perception of EMF risk. If people perceive themselves as less informed or actually possess less reliable knowledge, this can create uncertainty, which in turn may lead to an increased perception of risk. Further, differences in age or age structure of the professional groups should also be considered. Nursery school teachers are often younger than school teachers as a result of their professional training. It is possible that younger people use digital and alternative media more frequently on average, increasing the likelihood of encountering risky or non-evidence-based content. This could further influence EMF risk perception. At the same time, a definitive conclusion is not possible here, as data on the media use of nursery school teachers in Germany are lacking. Nonetheless, we cannot preclude some selection bias in the sense that nursery school teachers who use alternative media participated in the study in disproportionate numbers. This tendency could have biased our results towards an overestimation of the true risk perception in this group (see also further remarks on selection bias below).

Moreover, participants seemed to have interpreted the questions whether EMF health effects can also have non-physical causes differently. This is indicated by the results of the latent class analysis. Here, the class with the lowest risk perception (LC 1) had the highest levels both in agreement with the statement “Health effects caused by electromagnetic fields can also have non-physical causes” and in rejection of this statement. Accordingly, there does not seem to be a clear interpretation for this item.

One possible cause could have been the use of the word “non-physical”. It could be discussed whether participants perceived the statement as derogatory, as they might have understood “non-physical” to be synonymous with “not real” and therefore as imaginary, and consequently disagreed with the statement. Furthermore, it could be that some participants were of the opinion that complaints always have a non-physical component – agreement with this item could therefore be attributed to a general understanding of health – and not specifically to EMFs. It should also be taken into consideration that, due to possible problems in the interpretation of the item wording, agreement or disagreement may have resulted from uncertainty.

The heatmap, which compares the response combinations for the item on “EMF health effects below legal limits” and on “EMF health effects can also have non-physical causes”, showed that every response combination was represented. Furthermore, there was an increased frequency of disagreement and agreement with both statements. This seems plausible, as participants who believed, for example, that EMFs below the legal limits have no physical effect likely also understand this to mean that no psychosomatic effects are present, too. Conversely, participants who believed in the effects of EMF despite the legal limits probably cannot completely rule out psychosomatic effects. This could also explain the slightly higher level of agreement with the statement “EMF health effects can also have non-physical causes” compared to the item “EMF health effects below legal limits”. In addition, it is debatable whether participants were more likely to believe in psychosomatic than physical effects of EMF – this is at least supported by the frequently reported non-specific symptoms. It was very rare for participants to agree with the statement about health effects of EMF below legal limits while simultaneously rejecting the statement about non-physical causes. In contrast, the statement “EMF health effects below legal limits“ was rejected and the statement “EMF health effects can also have non-physical causes” supported in approximately 30% of cases. This could be due, firstly, to the difficulty of interpreting the non-physical causes statement. Secondly, participants might have assumed that the first question referred only to purely demonstrable, physical effects and therefore deny them, even though they are aware that some people develop symptoms due to their psychological state that are not considered “real” health effects of EMFs.

Altogether, EMFs appears to play only a little role in the everyday work of school teachers and nursery school teachers, as only between 14% and 16% of the participants reported that this topic came up at work. At the same time, participants indicated a very low subjective level of information regarding the topic of EMFs and health: about half of the school teachers and three-quarters of all participating nursery school teachers felt poorly informed. As mentioned before, EMFs and especially possible EMFs health effects just play a minor role in the studies/training of school teachers and nursery school teachers in Germany. This is also reflected in our results. In the focus groups, school teachers and nursery school teachers also communicated uncertainties regarding EMFs in general and expressed a clear need for scientifically sound, understandable, and practical information. Information about EMFs also rarely reached participants. Most of the school teachers and nursery school teachers did not search for information actively, and only a very small proportion received information passively. Accordingly, information on possible EMF health effects would need to be brought to them.

One consideration here would be the integration of topics relating to environmental health into university studies, training, or continuing education. Public risk communication institutions could provide target group-specific materials to educate the professional groups of school teachers and nursery school teachers about environmental aspects which could also include the topic of EMF exposure. Furthermore, short, concise, topic-specific informational videos from public offices or organisations would be beneficial. This would not only counteract false conclusions about EMFs based on missing knowledge or concern, but would also strengthen their role as multipliers of health-related information, as they could directly pass on scientifically accurate findings to concerned parents or young people when needed. Indirectly this also would influence pupils or very young children, because trustworthy, credible information would influence their caregivers’ attitudes towards EMFs.

### Compatibility with other studies

The present study was conducted exclusively among school and nursery school teachers without an external reference group such as the general population. We did not include a reference group from the general population for several reasons. First, the risk perception in the German general population towards EMF is already regularly measured by regular surveys of the Federal Office for Radiation Protection. Instead of providing additional, redundant data on the risk perception in the general population, the present study aimed at gaining a detailed understanding of not only the attitudes and beliefs towards EMFs specifically among school and nursery school teachers, but also on the information level and demands of these specific occupational groups. Consequently, the study questionnaire was drafted for these specific professional groups so that many questions would not have been applicable for individuals from the general population. Nonetheless, we acknowledge that it can be seen as a limitation that we could not perform direct analytical comparisons as to measure to degree to which school teachers differ in their risk perception concerning EMFs from the general population. We can only compare the results from the present study with the results from similar studies, taking into account that the instruments to assess EMF risk perception may differ between such other studies and our approach. In this context, it has to be mentioned that we used an almost identical questionnaire to measure attitudes and information level concerning EMFs in an earlier study among German physicians.

The survey among physicians using an almost identical methodology reported similar results for almost all items: here too, almost a third (28%) of the respondents agreed with the statement regarding EMF health effects below legal limits, while at the same time the subjective level of information on EMFs was also rather low as 60% of the physicians indicated that they felt poorly informed [38, 39]. Another, even earlier study among physicians from 2009 reported higher values of EMF concern among physicians compared to our survey [37]. However, this survey used a slightly different question to measure risk perception that did not specifically mention legal limits when asking whether participants believed that there are people who develop health issues as a consequence of EMF exposure. Besides, studies among physicians in Austria, the Netherlands, Switzerland, and France reported agreement rates between approximately 50 and 80% to questions implying that EMFs may cause health effects [60–63].

In 2024, a survey among a representative sample of the general population in the German-speaking region was conducted on behalf of the Federal Office for Radiation Protection. Risk perception regarding EMF was assessed in this study as well, even though with a slightly different question [64]. Here, 22% of participants indicated that they were (very) concerned about radiation from mobile phones and mobile phone base stations [64]. Furthermore, 19% stated that they were (very) concerned about radiation from high-voltage power lines, while a total of 17% indicated that they were (very) concerned about EMF in general [64].

Compared to the survey “What does Germany think about radiation?“, where one-fifth reported being very concerned regarding the radiation of smartphones, high voltage power lines, and mobile phone base stations [24], and compared to the representative telephone survey of the general population on EMF risk perception, school teachers and nursery school teachers in our study reported slightly higher EMF concerns. The reason for this difference could possibly be due to differences in the method of data collection, as the surveys among the general population were for example conducted via telephone interviews. Another possible explanation for the higher perceived risk of EMFs among these two professional groups could be the current rapid increase in the digitalisation of educational institutions. The proliferation of whiteboards in schools, for example, or the permanent availability of WiFi in kindergartens, could have led to heightened sensitivity regarding the topic of EMFs and health – especially if they have responsibility for children and at the same time the subjective level of information is low. Furthermore, the study population in “What Does Germany Think About Radiation?” reported that only about 40% felt well or very well informed by national radiation protection institutions [24]. This supports the findings of our study in which well over half of the school teachers and nursery school teachers felt poorly informed.

School teachers and nursery school teachers who expressed some concern regarding health risks related to EMFs most frequently cited mobile phone base stations, mobile phones, and WiFi/Bluetooth/computers as EMF sources relevant for health effects; this picture was also seen in the recent survey among physicians mentioned above [38, 39]. However, it must be emphasised that the results depend on the specific wording of the questions regarding EMFs and health. Furthermore, it has to be mentioned that physicians are a very different professional group from school teachers and nursery school teachers. A direct comparison of these two studies should therefore be treated with caution.

In the German survey conducted among the general population mentioned above, respondents were asked to rate potential sources of EMFs on a five-point scale [64]. The majority of respondents indicated that in their view EMFs emanate from mobile phones (63%), high-voltage or high-current power lines (62%), and mobile base stations (“antennas”) (60%) [64]. Approximately half of the respondents were aware that modems, routers, and signal boosters are sources of EMF (51%), as are Wi-Fi devices (49%) and household electrical appliances (46%) [64]. 46% cited the sun as a source of EMF, while devices such as PCs, laptops, televisions, and monitors were mentioned by 44% of the respondents. Furthermore, just under half of the respondents perceived a link between EMF and the occurrence of headaches/migraines (49%) and sleep disturbances (48%). A fairly high proportion also suspected a connection with concentration difficulties (41%), electrosensitivity (39%), the occurrence of brain tumors (35%), and other forms of cancer (33%) [64].

In our study, school teachers and nursery school teachers perceived the same sources of EMF exposure. The general population also cited the same symptoms in relation to EMF as school teachers and nursery school teachers; however, the proportion of school teachers and nursery school teachers who attributed these symptoms to EMF was slightly higher. This may be attributable to this professional group’s heightened perception of EMF-related risks. Nevertheless, the differences in the phrasing of the survey questions must also be taken into account here.

In addition, some of these studies were conducted many years ago, and risk perception regarding EMFs itself may have changed over time because some other issues – like for example concerns about climate change or antibiotics in food – are currently causing higher levels of concern compared to concerns regarding EMF exposure [39, 65, 66]. A study on modern health worries and sensitivity to electromagnetic fields from Belgium published in 2025 found that in 2020, alongside air pollution, pesticides in food, food additives, and pesticide sprays, mobile phone antennas were among the five most frequently named topics of concern [65]. However, by 2021, concerns about mobile phone antennas had already been superseded by concerns about climate change and the greenhouse effect [65]. The nationwide telephone survey among the general population in Germany also revealed that other the topics mentioned in the survey, e.g. multi-drug-resistant germs or nanoparticles and plastics, caused greater concern among a larger proportion of respondents than EMFs did [64].

Meanwhile, the perception of EMF risk might decrease over time, with studies suggesting that a habituation effect may occur or that the perceived benefits of EMF-generating technologies may outweigh the perceived risks [66, 67].

Five types of risk perception concerning electromagnetic fields (LC 1–LC 5) were identified from the study population using latent class analysis. These occurred with varying prevalence within the study population. Overall, approximately 11% of participants were classified as LC 5 and thus as very concerned. What is particularly striking about this group is the very high level of agreement regarding all symptoms and all EMF sources. These results can be linked to a study by Ledent et al., which examined the characteristics of people who report different levels of sensitivity to EMF [65]. This study reported that participants who indicated higher sensitivity to EMF had more concerns about it. These concerned participants also reported greater concerns about all the EMF sources mentioned in the study [65]. While our study did not explicitly ask whether participants were concerned about the EMF sources mentioned, it is nevertheless evident in LC 5 that the frequency of all the possible EMF sources mentioned is very high compared to groups 2 and 3, for example.

Furthermore, Ledent et al. found that participants who described themselves as EMF sensitive – and were accordingly more concerned about EMF – reported more nonspecific symptoms such as headaches, fatigue, or memory problems compared to non-sensitive participants [65]. In particular, our school teachers and nursery school teachers who were in LC 5 reported a very high incidence of possible symptoms triggered by EMF compared to the other latent classes. A particularly large number of participants from LC 5 believed that nonspecific symptoms such as headaches, sleep disturbances, restlessness, and fatigue could be caused by EMF.

### Strengths and limitations

The mixed-methods design used for this study allowed to gain in-depth insight into the topic of risk perception concerning EMF exposure and health through the combination of a quantitative survey and subsequent group discussions. This made the needs and options for action clearer.

Furthermore, the correction of prevalence estimates via Multilevel Regression and Poststratification based on high-quality data of the source population allowed to account for possible bias due to non-participation. Nonetheless, differences within the strata could not be resolved, i.e., if within the strata individuals with higher risk perception were overrepresented, we would still have an overestimation of the true risk perception among teachers that also MRP cannot address. Therefore, selection bias in risk perception estimates cannot be completely ruled out.

Since the selection and recruitment of participants was not probabilistic, it is uncertain how many school teachers and nursery school teachers were reached through the various forms of invitation. Hence, it was not possible to calculate a response rate so that the extent of a possible selection bias cannot be estimated. Consequently, we cannot preclude that the true EMF risk perception among German school teachers and nursery school teachers may have been overestimated due to the increased participation of EMF-concerned individuals. At the same time, the small changes in the prevalence estimates after correction for non-response using MRP and the high comparability of the study and source populations with respect to socio-demographic characteristics speak against a large degree of bias due to non-response.

Besides, we cannot be entirely sure that every participant actually belonged to the professional group of school teachers and nursery school teachers. Although the study invitation and description explicitly mentioned the target group of school teachers and nursery school teachers, the study advertisement was posted exclusively in relevant groups on social media, and only selected federal and state associations of the professional groups were contacted for the study; unauthorised access to the online questionnaire cannot be completely ruled out. In fact, at the beginning of the field phase, we received a considerable number of questionnaires that were apparently completed by bots. By explicitly excluding bots (for example, due to exclusion of questionnaires with a very short completion time of less than 5 min) and by introducing a captcha test before the start of the survey, the influence of bots was minimised as much as possible, but cannot be completely ruled out. However, the sensitivity analyses suggest that there were no relevant differences in the results between participants invited by mail and those recruited online. Only the prevalence estimates for subjective information levels show some differences between the complete study population and the study population that was invited by post. The subjective level of information regarding EMF among the participants who were invited by post was lower for both teachers and nursery school teachers. It could be discussed here whether the participants contacted via post exhibit different levels of digital health literacy and internet usage compared to those contacted online. Those contacted online might have more frequent access to information regarding EMF and consequently feel somewhat better informed – we refer here to, among other things, frequently used information sources regarding EMF: websites of public organisations and social media posts. Furthermore, as already mentioned, despite extensive countermeasures, it cannot be completely ruled out that the results of the online recruited participants were influenced by responses from fake accounts.

Another constraint of our study is that it was not grounded in a specific theoretical framework. Instead, it had a rather exploratory character aiming at gaining an initial insight into teachers’ attitudes and beliefs towards EMFs and health. In order to develop a more nuanced understanding of EMF risk perception among teachers, future research may apply a strong theoretical foundation such as the Psychometric Paradigm [68, 69] or Protection Motivation Theory [70] to derive and examine specific hypotheses, possibly also based on the initial findings provided by the present study. Furthermore, we need to highlight that even though our questionnaire was based on the tools used in previous, comparable surveys [37–39], it has not been validated by assessing its psychometric properties, e.g. with respect to factor structure. Developing a rigorously tested and validated instruments for measuring EMF-related risk perception by means of methods like exploratory and confirmatory factor analysis may therefore be a worthwhile objective for future research in the field.

As previously mentioned, the absence of a reference group did not allow to directly compare school teachers and nursery school teachers with the general population. Consequently, external validity is limited, and it remains unclear whether the observed EMF risk perception among our target group is specific to this group or if it occurs in a similar manner within the general population. Furthermore, this complicates the assessment of the magnitude of the associations identified. Nevertheless, it should be emphasised once again that this study is primarily exploratory in nature, aiming to gain initial insights into the attitudes and beliefs of teaching staff regarding electromagnetic fields and health. Future studies should definitely incorporate comparison groups to facilitate better contextualisation and generalization of the results.

Concerning the LCA, it needs to be highlighted that two of the selected indicator variables (“health effects” and “EMF sources”) were multiple-choice items where the likelihood of selecting one option may depend from the likelihood of selecting another option. Especially in the case of the “health effects” variable, selecting one non-specific symptom (e.g., headaches) may have increased the probability of selection other non-specific symptoms (e.g., vertigo) as well. This may have compromised the fulfilment of the local independence assumption as an important LCA criterion. Furthermore, even though our indicator variables fulfilled the measurement level criterion of indicators to be categorical variables, two of them (“EMF health effects below legal limits” and “EMF health effects can also have non-physical causes”) entered the LCA as binary variables that were derived from 5-point individual Likert items. Why this approach of dichotomising a 5-point item should not have violated the measurement level criterion, the resulting change in the probability structure of the items from ordinal to binomial needs to be mentioned. Furthermore, the dichotomisation may have led to some loss of information, e.g. with respect to the intensity of attitudes towards EMFs.

### Implications

Overall, there seems to be a need for scientifically sound, understandable, and practical information regarding EMFs and health among school teachers and nursery school teachers. The qualitative discussions implied that the lack of professional discussion was attributed to a lack of continuing education opportunities and the absence of professional discourse. Since a lack of information can lead to uncertainty and incorrect conclusions, it is particularly important to provide targeted education to multiplier groups such as school teachers and nursery school teachers. For this purpose, tailored communication formats for informing this target group may be developed, as these are currently lacking (at least in Germany). Brochures or flyers with short, concisely written, modularly structured, and graphically modernised text passages would be conceivable here. Furthermore, audio-visual formats on the topic of EMFs and health in the form of short videos, provided, for instance, by public bodies such as radiation protection authorities, could enhance knowledge levels.

At the same time, one may argue that what is much more sustainable is to foster the overall scientific literacy in the general population in order to help members of the public to better understand and critically appraise scientific literature. This appears all the more important as public trust in science, particularly health science, is declining, with potentially very negative consequences for public health and evidence-based health policies [71, 72]. To counteract this, besides strengthening science education during the educational career of children and adolescents (i.e., in schools), various additional approaches to enhance scientific literacy in the general population are discussed in the literature. These include scientist outreach or ambassador programmes as well as initiatives to train scholars how to communicate their research in a way that is interesting and understandable to lay persons [73, 74]. This may also encompass formal education in science communication, ideally already during undergraduate training [75]. Other argue that researchers and health authorities may double-check their strategies to reach and address the public, possibly adapting innovative communication strategies [76]. A promising approach in this context may be to collaborate with (trustworthy) “medical influencer” who are skilled in communicating accurate scientific information to their followers [76]. We hope that the present manuscript can provide some helpful information for stakeholders in the field of EMFs how to adapt their communication and outreach strategies to professional groups like teachers who are acting as multipliers to the general population.

## Conclusion

A notable proportion of school and nursery school teachers indicated that in their view EMF exposure below legal limits may damage human health. At the same time, most teachers considered themselves as poorly informed about this topic. Given their role as influential multipliers, this lack of information carries the risk of disseminating inaccurate or incorrect information among the public. Consequently, there is a need for comprehensible and evidence-based information formats and strategies tailored to this target group.

## Supplementary Information

Supplementary Material 1.

Supplementary Material 2.

## Data Availability

The data used in the present study is not available from the authors due to data protection.

## Abbreviations

CI: Confidence Interval
EMFs: Electromagnetic Fields
ICNIRP: International Commission on Non-Ionizing Radiation Protection
LCA: Latent class analysis
MRP: Multilevel Regression and Poststratification
