# Supplementary material for: Risk perception of electromagnetic fields among school teachers and nursery school teachers: a mixed-methods study
Source: BMC Public Health. 2026 Jun 23;26:2117. doi: 10.1186/s12889-026-27964-3 (PMC13352703; doi:10.1186/s12889-026-27964-3)
Supplement: Supplementary file 1 — Supplementary Material 1. [file 12889_2026_27964_MOESM1_ESM.docx]

# Additional file 1

## Questionnaire

The original questionnaire is in German (below). The questionnaire was only translated to English for presentation in this additional file. German questions were translated by one person without back-translating and should not be used without further validation.

### English Version

# Demographics and working environment

**What professional title applies to you?**

- Teacher
- Educator
- Other ____________

**How old are you?**

- ≤ 20 years
- 20-29 years
- 30-39 years
- 40-49 years
- 50-59 years
- ≥ 60 years

**What is your sex?**

- Female
- Male
- Diverse

**The sponsorship of the institution where you primary work is…**

- Public
- Private
- Other ___________

**In which type of school do you teach most often? (This question is only displayed to teachers.) (Note: The exact terms may vary depending on the federal state.)**

- Elementary School
- Secondary/Middle School
- Secondary Modern School
- Grammar School
- Comprehensive School
- School type with multiple educational exits
- Free Waldorf School
- Special school
- Further Education Colleges
- Others ________

**Which school subjects do you teach? (This question is only displayed to teachers)**

_____________________________________________________________________

**What age are the children you mainly work with (This question is only displayed to educators.)**

- ≤ 3 years
- 3–6 years
- 7–10 years
- 11–14 years
- ≥15 years

**Does the institution where you mainly work have any other special features that you would like to mention here (e.g., a specific educational concept such as Montessori, etc.)?**

_____________________________________________________________________

**In which federal state do you mainly work?**

- Baden-Württemberg
- Bavaria
- Berlin
- Brandenburg
- Bremen
- Hamburg
- Hesse
- Mecklenburg-Western Pomerania
- Lower Saxony
- North Rhine-Westphalia
- Rhineland-Palatinate
- Saarland
- Saxony
- Saxony-Anhalt
- Schleswig-Holstein
- Thuringia

**How many inhabitants does the town have where the facility where you primarily work is located?**

- < 5.000
- 5.000–10.000
- 10.000–20.000
- 20.000–100.000
- > 100.000

# Risk perception

Note: The following questions regarding possible health effects of electromagnetic fields always assume compliance with the legally defined limits.

**To what extent do you agree with the following statements?**

| Disagree |  |  |  | Agree |
| --- | --- | --- | --- | --- |
| \|__\| | \|__\| | \|__\| | \|__\| | \|__\| |

**“There are individuals who develop adverse health effects from electromagnetic fields below legal limits.“**

**“Adverse health effects from electromagnetic fields can also have non-physical causes.“**

| Disagree |  |  |  | Agree |
| --- | --- | --- | --- | --- |
| \|__\| | \|__\| | \|__\| | \|__\| | \|__\| |

# Sources of electromagnetic fields and their effects

**In your opinion, which adverse health effects can be caused by electromagnetic fields? (Multiple answers are possible)**

- Headaches
- Sleep disorders
- Nervousness / restlessness
- Difficulties concentrating
- ADHD / behavioral disorders
- Vertigo
- Tinnitus / hearing disorder
- Visual disorders
- Fatigue
- Cardiac arrhythmia
- Cancer
- Alzheimer's disease
- Other _______________

**In your opinion, which sources produce electromagnetic fields that can cause adverse health effects? (Multiple answers are possible)**

- Cell phones
- Cell phone base stations
- Cordless landline telephones
- Radio / TV
- WiFi / Bluetooth / computers
- Microwave
- Induction cooker
- Power lines
- Digital boards / Whiteboards
- Other _______________

# Electromagnetic fields in everyday working life

**Have adverse health effects due to electromagnetic fields ever been discussed at work?**

- Yes
- No

**On what occasion did the health effects of EMF come up in your professional life? (Question was only displayed if 'yes' was previously selected.)**

- As part of lessons
- At parents’ evenings
- During discussions with parents/guardians
- During discussions with colleagues
- when children/young people raise the topic
- Other _______________

**How often have health effects due to electromagnetic fields come up in your working life during the last 12 months? (Question was only displayed if 'yes' was previously selected.)**

- 0 times
- 1-4 times
- 5-9 times
- 10-49 times
- 50-99 times
- ≥ 100 times

# Level of information and information requests

**How well do you feel informed about potential health effects of electromagnetic fields?**

| Very poorly |  |  |  | Very well |
| --- | --- | --- | --- | --- |
| \|__\| | \|__\| | \|__\| | \|__\| | \|__\| |

**Have you received any new information about the health effects of EMF during the past 12 months? (Multiple answers can be selected.)**

- Yes, I searched actively for information
- Yes, I received information randomly/passively
- No, I neither searched for information actively nor did I receive information by chance

**Which sources had provided you these information during the past 12 month? (Multiple answers are possible)**

- Public broadcasting (Radio, TV, internet services, e.g., Tagesschau or tageschau.de).
- Private broadcasting (Radio, TV, internet services, e.g., RTL aktuell oder RTL.de).
- Local or regional tabloid newspapers (e.g., Kölner Express, B.Z.).
- National tabloid newspapers (e.g., Bild or bild.de).
- Local or regional quality newspapers (e.g., Hannoversche Allgemeine Zeitung, Osnabrücker Zeitung).
- National quality newspapers or news magazines (e.g., FAZ, faz.net, Spiegel, spiegel.de).
- Social media posts (e.g., via Facebook, Telegram).
- Comments of internet users (e.g., in forums, blogs, or comment sections).
- Alternative media (e.g., Russia Today, Ken FM, Tichys Einblick, Achse des Guten, Reitschuster).
- Websites of public organisations (e.g., Federal Office for Radiation Protection, World Health Organization).
- Articles in scientific journals (e.g., PubMed).
- Other information sources: _______________

**Regarding which aspects of health effects due to electromagnetic fields would you like more information?**

________________________________________________________________________

### German Version

# Demographie und Arbeitsumfeld

**Welche Berufsbezeichnung trifft auf Sie zu?**

- Lehrer*in
- Erzieher*in
- Andere, bitte eintragen: _____________________

**Wie alt sind Sie?**

- Unter 20 Jahren
- 20-29 Jahre
- 30-39 Jahre
- 40-49 Jahre
- 50-59 Jahre
- 60 Jahre und älter

**Welchem Geschlecht fühlen Sie sich zugehörig?**

- Weiblich
- Männlich
- Divers

**Die Trägerschaft der Einrichtung, in der Sie hauptsächlich arbeiten, ist...**

- Öffentlich
- Privat
- Andere Trägerschaft, und zwar ___________

**In welcher Schulform unterrichten Sie mehrheitlich? (Diese Frage wird nur Lehrer*innen angezeigt) (Hinweis: Die genauen Bezeichnungen können je nach Bundesland abweichen.)**

- Grundschule
- Hauptschule/Mittelschule
- Realschule
- Gymnasium
- Integrierte Gesamtschule
- Schulart mit mehreren Bildungsabgängen
- Freie Waldorfschule
- Förderschule
- Berufliche Schule (z. B. Berufschule, FOS, BOS, Fachschule)
- Andere Schulform: _____________________

**Welche Schulfächer unterrichten Sie? (Diese Frage wird nur Lehrer*innen angezeigt)**_____________________________________________________________________

**Welches Alter haben die Kinder, mit denen Sie hauptsächlich arbeiten? Mehrere Antworten können ausgewählt werden (Diese Frage wird nur Erzieher*innen angezeigt)**

- Unter 3 Jahren
- 3-6 Jahre
- 7-10 Jahre
- 11-14 jahre
- 15 Jahre und älter

**Weist die Einrichtung, in der Sie hauptsächlich arbeiten, weitere Besonderheiten auf, die Sie hier gerne nennen möchten (z. B. ein spezielles pädagogisches Konzept, wie Montessori, etc.)?**

_____________________________________________________________________

**In welchem Bundesland arbeiten Sie hauptsächlich?**

- Baden-Württemberg
- Bayern
- Berlin
- Brandenburg
- Bremen
- Hamburg
- Hessen
- Mecklenburg-Vorpommern
- Niedersachsen
- Nordrhein-Westfalen
- Rheinland-Pfalz
- Saarland
- Sachsen
- Sachsen-Anhalt
- Schleswig-Holstein
- Thüringen

**Wie viele Einwohner*innen hat der Ort, in dem sich die Einrichtung, in der Sie hauptsächlicharbeiten, befindet?**

- < 5.000
- 5.000–10.000
- 10.000–20.000
- 20.000–100.000
- > 100.000

# Risikowahrnehmung

Hinweis: Die nachfolgenden Fragen zu möglichen Gesundheitsbeschwerden durch elektromagnetische Felder setzen immer voraus, dass die Einhaltung der gesetzlich festgelegten Grenzwerte gegeben ist.

**Inwieweit stimmen Sie den folgenden Aussagen zu?**

**„Es gibt Personen, bei denen unter Einhaltung der gesetzlichen Grenzwerte Gesundheitsbeschwerden durch elektromagnetische Felder ausgelöst werden.“**

| Stimme nicht zu |  |  |  | Stimme zu |
| --- | --- | --- | --- | --- |
| \|__\| | \|__\| | \|__\| | \|__\| | \|__\| |

**„Gesundheitsbeschwerden durch elektromagnetische Felder können auch nicht-körperliche Ursachen haben.“**

| Stimme nicht zu |  |  |  | Stimme zu |
| --- | --- | --- | --- | --- |
| \|__\| | \|__\| | \|__\| | \|__\| | \|__\| |

# Quellen elektromagnetischer Felder und deren Auswirkung

**Welche Gesundheitsbeschwerden können Ihrer Einschätzung nach durch elektromagnetische Felder ausgelöst werden? (Mehrere Antworten sind möglich)**

- Kopfschmerzen
- Schlafstörungen
- Nervosität / Unruhe
- Konzentrationsstörungen
- ADHS / Verhaltensauffälligkeiten
- Schwindel
- Tinnitus / Hörstörungen
- Sehstörungen
- Abgeschlagenheit / Müdigkeit
- Herzrhythmusstörungen
- Krebserkrankungen
- Alzheimer
- Sonstige _______________

**Welche Quellen produzieren Ihrer Einschätzung nach elektromagnetische Felder, die Gesundheitsbeschwerden auslösen können? (Mehrere Antworten sind möglich)**

- Mobiltelefone
- Mobilfunkbasisstationen („Handymast“)
- Schnurlose Festnetztelefone
- Radio / Fernsehen
- WLAN / Bluetooth / Computer
- Mikrowellenkochgeräte
- Induktionsherd
- Hochspannungsleitungen
- Digitale Tafeln / Whiteboards
- Sonstige _______________

# Elektromagnetische Felder im Berufsalltag

**Sind in Ihrem Berufsalltag schon einmal gesundheitliche Wirkungen elektromagnetischer Felder zur Sprache gekommen?**

- Ja
- Nein

**Bei welcher Gelegenheit kam die Thematik gesundheitlicher Wirkungen elektromagnetischer Felder in Ihrem Berufsalltag zur Sprache? (Mehrere Antworten können ausgewählt werden) (Frage wird nur angezeigt, wenn zuvor ‚ja‘ angekreuzt wurde)**

- Im Rahmen des Unterrichts
- An Elternabenden
- Bei Gesprächen/Sprechstunden mit Eltern/Erziehungsberechtigten
- Bei Gesprächen mit Arbeitskolleg*innen
- Kinder/Jugendliche haben das Thema angesprochen
- Andere Gelegenheit, und zwar: _______________

**Wie oft sind während der letzten 12 Monate in Ihrem Berufsalltag gesundheitliche Wirkungen elektromagnetischer Felder zur Sprache gekommen? (Frage wird nur angezeigt, wenn zuvor ‚ja‘ angekreuzt wurde)**

- 0 Mal
- 1-4 Mal
- 5-9 Mal
- 10-49 Mal
- 50-99 Mal
- ≥ 100 Mal

# Informationswünsche und Informationsstand

**Wie gut fühlen Sie sich über mögliche gesundheitliche Wirkungen elektromagnetischer Felder informiert?**

| Sehr schlecht |  |  |  | Sehr gut |
| --- | --- | --- | --- | --- |
| \|__\| | \|__\| | \|__\| | \|__\| | \|__\| |

**Haben Sie während der vergangenen 12 Monate neue Informationen zu gesundheitlichen Wirkungen elektromagnetischer Felder erhalten? (Mehrere Antworten können ausgewählt werden)**

- Ja, ich habe aktiv nach Informationen gesucht.
- Ja, ich bin zufällig/passiv auf Informationen gestoßen (z. B. durch Radiobeiträge, Artikel in Zeitschriften, etc.).
- Nein, ich habe weder aktiv nach Informationen gesucht, noch bin ich zufällig auf Informationen gestoßen.

**Über welche Quellen haben Sie diese Informationen erhalten? (Mehrere Antworten können ausgewählt werden)**

- Durch den öffentlich-rechtlichen Rundfunk (Radio, Fernsehen, Internetangebote, z. B. Tagesschau oder tageschau.de).
- Durch den privaten Rundfunk (Radio, Fernsehen, Internetangebote, z. B. RTL aktuell oder RTL.de).
- Durch lokale oder regionale Boulevardzeitungen (z. B. Kölner Express, B.Z.).
- Durch überregionale Boulevardzeitungen (z. B. Bild oder bild.de).
- Durch lokale oder regionale Qualitätszeitungen (z. B. Hannoversche Allgemeine Zeitung, Osnabrücker Zeitung).
- Durch überregionale Qualitätszeitungen oder Nachrichtenmagazine (z. B. FAZ, faz.net, Spiegel, spiegel.de).
- Durch Beiträge, die andere Nutzer*innen in sozialen Netzwerken teilen (z.B. Facebook, Telegram).
- Durch Beiträge oder Kommentare von Internetnutzer*innen (z. B. in Foren, Blogs, Kommentarbereichen).
- Durch alternative Medien (z. B. AUF1, Tichys Einblick, Achse des Guten, Reitschuster).
- Auf Internetseiten öffentlicher Organisationen (z. B. Bundesamt für Strahlenschutz, Weltgesundheitsorganisation).
- Durch Artikel in wissenschaftlichen Fachzeitschriften
- Ich nutze sonstige Informationsquellen, und zwar: _____________

**Zu welchen Aspekten gesundheitlicher Wirkungen elektromagnetischer Felder wünschen Sie sich weitere Informationen?**

_____________________________________________________________________
