# Supplementary material for: Risk perception of electromagnetic fields among school teachers and nursery school teachers: a mixed-methods study
Source: BMC Public Health. 2026 Jun 23;26:2117. doi: 10.1186/s12889-026-27964-3 (PMC13352703; doi:10.1186/s12889-026-27964-3)
Supplement: Supplementary file 2 — Supplementary Material 2. [file 12889_2026_27964_MOESM2_ESM.docx]

## Supplement

S 1: Descriptive values of the variable ‘EMF health effects below legal limits’ and ‘EMF health effects can also have non-physical causes’

| **Variable** |  | **I do not agree** |  |  |  | **I agree** |
| --- | --- | --- | --- | --- | --- | --- |
|  | Missing | 0 | 1 | 2 | 3 | 4 |
| **EMF health effects below legal limits** | 1 (0.1%) | 379 (27.1%) | 262 (18.7%) | 325 (23.2%) | 206 (14.7%) | 227 (16.2%) |
| **EMF health effects can also have non-physical causes** | 6 (0.4%) | 294 (21.1%) | 181 (13.0%) | 339 (24.3%) | 251 (18.0%) | 329 (23.6%) |

N EMF health effects below legal limits = 1399,

N EMF health effects can also have non-physical causes = 1394

S 2: Sensitivity analysis of the variable " EMF health effects below legal limits ", where the middle category of the Likert scale was considered agreement; proportion of participants who agreed with the statement; by professional group

| Study population | Empirical | Corrected |
| --- | --- | --- |
| **Total** | 54.2% (51.6%-56.8%) | - |
| **School teachers total** | 51.7% (48.6%-54.7%) | 50.8% (46.2%-54.9%) |
| Female school teachers | 59.7% (55.9%-63.5%) | 56.4% (51.0%-61.8%) |
| Male school teachers | 37.6% (32.7%-42.6%) | 36.7% (31.6%-42.4%) |
| **Nursery school teachers total** | 62.3% (57.0%-67.5%) | 65.8% (60.4%-71.2%) |
| Female nursery school teachers | 67.2% (61.4%-72.9%) | 67.8% (61.8%-73.6%) |
| Male nursery school teachers | 44.3% (32.6%-55.9%) | 43.8% (32.3%-55.2%) |

S 3: Parameter estimates including 95% confidence intervals for the model parameters of the latent class analysis; the numbers correspond to the values in Figure 5; gray: values <10%, bold: values >50%

| Indikator | LC 1 | LC 2 | LC 3 | LC 4 | LC 5 |
| --- | --- | --- | --- | --- | --- |
|  | low risk perception | rather low risk perception regarding selected EMF sources and selected health problems | medium to rather high risk perception regarding selected EMF sources and selected health problems | people with medium to rather high risk perception regarding all EMF sources surveyed and selected health complaints | high risk perception regarding all EMF sources and all health complaints |
| **Prevalence of latent classes** | | | | | |
|  | 27.7%  (25.3-30.0%) | 24.7%  (21.9-27.5%) | 29.9%  (26.7-33.1%) | 7.3% (5.2%-9.3%) | 10.5% (8.7%-12.3%) |
| **Response probabilities by class** | | | | | |
| **There are individuals who develop adverse health effects from EMF below legal limits** | | | | | |
| I do not agree (0) | **98.0% (96.3-99.7%)** | 0.0% (0.0-0.0%) | 0.0% (0.0-0.0%) | 0.0% (0.0-0.0%) | 0.0% (0.0-0.0%) |
| 1 | 0.5% (-0.3-1.3%) | **53.6% (46.9-60.2%)** | 11.7% (7.8-15.5%) | 16.5% (5.7-27.2%) | 6.3% (1.3-11.3%) |
| 2 | 1.5% (-0.1-3.0%) | 31.3% (25.3-37.3%) | 32.2% (26.6-37.9%) | 39.0% (25.2-52.8%) | 24.9% (15.8-34.0%) |
| 3 | 0.0% (0.0-0.0%) | 11.6% (7.4-15.8%) | 26.9% (21.9-31.9%) | 23.8% (12.3-35.2%) | 20.0% (11.4-28.6%) |
| I agree (4) | 0.0% (0.0-0.0%) | 3.5% (1.0-6.0%) | 29.2% (23.5-34.9%) | 20.8% (8.6-32.9%) | 48.9% (37.4-60.3%) |
| **Adverse health effects from EMF can also have non-physical causes** | | | | | |
| I do not agree  (0) | **54.8% (49.8-59.9%)** | 12.3% (8.4-16.3%) | 5.5% (2.9-8.1%) | 9.3% (1.7-16.8%) | 5.6% (1.3-10.0%) |
| 1 | 5.9% (3.6-8.3%) | 30.4% (24.6-36.3%) | 6.7% (3.7-9.7%) | 15.9% (4.8-27.0%) | 6.2% (1.0-11.3%) |
| 2 | 8.6% (5.6-11.5%) | 29.3% (23.5-35.2%) | 29.3% (23.9-34.8%) | 38.6% (25.6-51.7%) | 29.6% (20.4-38.8%) |
| 3 | 6.0% (3.6-8.4%) | 19.4% (14.4-24.3%) | 29.3% (24.0-34.6%) | 16.1% (6.6-25.7%) | 15.7% (7.7-23.6%) |
| I agree (4) | 24.6% (20.3-29.0%) | 8.5% (5.0-12.1%) | 29.2% (23.5-34.9%) | 20.1% (8.4-31.7%) | 43.0% (32.3-53.6%) |
| **EMF sources** | | | | | |
| Mobile phones | 0.0% (0.0-0.0%) | **67.8% (61.9-73.7%)** | **91.0% (87.6-94.4%)** | **100.0% (100-100%)** | **100.0% (100-100%)** |
| Mobile phone base stations | 0.0% (0.0-0.0%) | **53.8% (47.3-60.3%)** | **79.7% (74.9-84.5%)** | **96.5% (91.1-102.0%)** | **94.0% (88.8-99.2%)** |
| Cordless landline telephones | 0.0% (0.0-0.0%) | 22.0% (16.7-27.3%) | 37.1% (31.3-43.0%) | **90.6% (80.9-100.2%)** | **79.7% (70.5-88.9%)** |
| Radio / Television | 0.0% (0.0-0.0%) | 10.0% (6.0-14.0%) | 16.8% (12.2-21.3%) | **58.5% (41.5-75.4%)** | **62.1% (52.3-71.8%)** |
| WiFi / Bluetooth / Computer | 0.0% (0.0-0.0%) | 43.6% (37.1-50.1%) | **83.6% (79.2-88.0%)** | **97.4% (92.6-102.3%)** | **96.1% (92.0-100.2%)** |
| Microwave | 0.2% (-0.3-0.8%) | 17.5% (12.6-22.3%) | 38.9% (33.2-44.6%) | **85.6% (73.7-97.4%)** | **81.6% (73.8-89.4%)** |
| Induction cooker | 0.0% (0.0-0.0%) | 12.6% (8.4-16.9%) | 13.0% (8.9-17.2%) | **73.2% (58.6-87.8%)** | **54.3% (43.4-65.2%)** |
| Power lines | 0.0% (0.0-0.0%) | 42.0% (35.5-48.5%) | 46.6% (40.5-52.6%) | **86.8% (77.1-96.4%)** | **82.0% (73.6-90.4%)** |
| Digital boards / Whiteboards | 0.0% (0.0-0.0%) | 8.5% (5.0-12.0%) | 28.5% (23.3-33.7%) | **61.6% (45.7-77.5%)** | **73.4% (64.5-82.2%)** |
| **Health complains** | | | | | |
| Headaches | 0.0% (0.0-0.0%) | **73.2% (67.6-78.7%)** | **95.9% (93.4-98.4%)** | **83.6% (73.7-93.6%)** | **99.3% (97.8-100.9%)** |
| Sleeping disorders | 0.0% (0.0-0.0%) | **58.0% (51.8-64.2%)** | **93.1% (90.2-96.1%)** | **87.8% (78.1-97.4%)** | **100.0% (100-100%)** |
| Nervousness / Restlessness | 0.0% (0.0-0.0%) | 41.9% (35.6-48.3%) | **85.2% (80.7-89.6%)** | **57.9% (43.4-72.3%)** | **100.0% (100-100%)** |
| Difficulties concentrating | 0.0% (0.0-0.0%) | 37.0% (30.9-43.2%) | **87.6% (83.6-91.6%)** | **69.5% (55.7-83.3%)** | **100.0% (100-100%)** |
| ADHD / Behavioural problems | 0.0% (0.0-0.0%) | 7.5% (4.0-10.9%) | 24.2% (19.4-29.0%) | 13.1% (3.6-22.6%) | **71.1% (61.2-80.9%)** |
| Vertigo | 0.0% (0.0-0.0%) | 14.3% (9.9-18.7%) | 36.8% (31.2-42.5%) | 10.1% (0.4-19.7%) | **89.1% (82.1-96.2%)** |
| Tinnitus / Hearing disorders | 0.0% (0.0-0.0%) | 10.7% (6.9-14.5%) | 23.8% (18.8-28.8%) | 15.9% (5.1-26.8%) | **86.1% (78.3-93.9%)** |
| Vision disorders | 0.2% (-0.3-0.8%) | 10.6% (6.6-14.7%) | 24.7% (19.7-29.7%) | 14.0% (4.2-23.8%) | **77.3% (69.0-85.7%)** |
| Fatigue | 0.0% (0.0-0.0%) | 15.0% (10.4-19.6%) | **63.9% (58.2-69.5%)** | **50.9% (35.7-66.2%)** | **95.8% (91.8-99.9%)** |
| Cardiac arrhythmias | 0.0% (0.0-0.0%) | 13.5% (9.1-17.8%) | 37.2% (31.7-42.7%) | 31.8% (19.2-44.3%) | **79.3% (71.1-87.6%)** |
| Cancer | 0.0% (0.0-0.0%) | 10.5% (6.7-14.4%) | 19.4% (14.7-24.1%) | 29.7% (17.3-42.2%) | **66.1% (55.1-77.2%)** |
| Alzheimer’s disease | 0.0% (0.0-0.0%) | 1.6% (-0.1-3.2%) | 5.1% (2.6-7.6%) | 1.3% (-1.9-4.4%) | 41.1% (30.5-51.8%) |

S 4: Description of the study population according to latent class membership. LC: latent class. For columns LC 1 to LC 5, relative frequencies (%), means, and standard deviations (SD) were averaged across the 20 assignments and presented together with the 5% and 95% percentiles (in parentheses)

| **Variable** | **Missing values** | **Total** | **LC 1** | **LC 2** | **LC 3** | **LC 4** | **LC 5** |
| --- | --- | --- | --- | --- | --- | --- | --- |
|  | **n (%)** | **n (%)** | **%** | **%** | **%** | **%** | **%** |
| **Professional group** | | | | | | | |
| School teachers | 0 (0.0) | 1053 (75.2) | 80.4 (80.3-80.6) | 74.1 (73.0-74.9) | 72.8 (71.7-74.0) | 79.4 (75.9-83.2) | 68.2 (66.4-70.0) |
| Nursery school teachers |  | 329 (23.5) | 18.5 (18.3-18.7) | 23.9 (23.1-24.9) | 26.1 (24.8-27.1) | 20.3 (16.8-23.3) | 30.4 (28.6-32.0) |
| Others |  | 18 (1.3) | 1.0 (1.0-1.0) | 2.1 (1.7-2.6) | 1.1 (0.5-1.4) | 0.3 (0.0-1.1) | 1.4 (1.3-1.9) |
| **Age group** | | | | | | | |
| < 30 years | 0 (0.0) | 183 (13.1) | 11.9 (11.8-11.9) | 11.5 (10.8-12.1) | 14.0 (13.3-14.9) | 13.8 (11.6-17.0) | 16.5 (14.9-18.1) |
| 30-39 years |  | 441 (31.5) | 32.8 (32.7-32.9) | 35.9 (34.7-36.8) | 29.4 (28.0-31.2) | 25.7 (21.4-29.8) | 27.5 (24.0-29.7) |
| 40-49 years |  | 391 (27.9) | 28.2 (28.0-28.4) | 27.7 (26.5-29.6) | 28.6 (26.7-30.0) | 24.2 (21.6-26.2) | 28.4 (26.2-29.8) |
| 50-59 years |  | 288 (20.6) | 19.6 (19.4-19.7) | 17.4 (16.3-18.6) | 21.6 (20.6-22.6) | 30.3 (27.7-34.4) | 21.4 (19.4-22.8) |
| ≥ 60 years |  | 97 (6.9) | 7.5 (7.5-7.5) | 7.5 (6.9-8.4) | 6.4 (5.4-7.1) | 6.0 (4.4-9.6) | 6.2 (5.3-7.5) |
| **Gender** | | | | | | | |
| Male | 10 (0.7) | 459 (33.0) | 46.7 (46.5-46.9) | 37.8 (36.4-39.3) | 22.5 (20.4-24.1) | 26.0 (22.7-29.3) | 20.6 (18.3-22.5) |
| Female |  | 931 (67.0) | 53.3 (53.1-53.5) | 62.2 (60.7-63.6) | 77.5 (75.9-79.6) | 74.0 (70.7-77.3) | 79.4 (77.5-81.7) |
| **Sponsorship** | | | | | | | |
| Public | 0 (0.0) | 1163 (83.1) | 86.6 (86.5-86.8) | 81.7 (80.8-82.7) | 81.4 (80.0-82.7) | 87.7 (85.6-90.2) | 78.4 (77.0-79.5) |
| Private |  | 237 (16.9) | 13.4 (13.2-13.5) | 18.3 (17.3-19.2) | 18.6 (17.3-20.0) | 12.3 (9.8-14.4) | 21.6 (20.5-23.0) |
| **Type of municipality** | | | | | | | |
| < 5,000 inhabitants | 2 (0.1) | 170 (12.2) | 11.3 (11.1-11.4) | 13.5 (12.6-14.5) | 13.0 (11.9-14.1) | 10.9 (8.7-13.8) | 9.8 (8.6-11.3) |
| 5,000-10,000 inhabitants |  | 210 (15.0) | 11.6 (11.6-11.7) | 14.9 (13.8-15.9) | 17.8 (16.8-18.8) | 12.1 (9.3-14.4) | 18.3 (16.6-20.0) |
| 10,000-20,000 inhabitants |  | 303 (21.7) | 20.4 (20.3-20.5) | 22.0 (20.8-23.3) | 20.0 (18.4-21.4) | 28.0 (26.4-29.6) | 24.7 (22.6-26.7) |
| 20,000-100,000 inhabitants |  | 353 (25.3) | 25.5 (25.3-25.6) | 24.0 (22.8-24.9) | 25.7 (24.5-26.6) | 23.3 (19.9-26.8) | 27.7 (26.1-29.5) |
| > 100,000 inhabitants |  | 362 (25.9) | 31.2 (31.0-31.4) | 25.5 (24.6-26.9) | 23.6 (22.4-24.8) | 25.7 (23.0-31.0) | 19.6 (17.9-21.6) |
| **Relevance of EMF in everyday working life** | | | | | | | |
| During the past year | 43 (3.1) | 239 (17.6) | 12.4 (12.4-12.5) | 17.1 (16.2-18.3) | 19.6 (18.6-21.1) | 22.4 (17.4-26.1) | 24.0 (23.0-25.6) |
| **Subjective information level** | | | | | | | |
| Poor | 77 (5.5) | 828 (62.6) | 42.3 (42.2-42.5) | 61.3 (60.2-62.3) | 76.3 (74.8-77.6) | 74.0 (70.3-77.3) | 73.2 (71.3-75.5) |
| **Reception of new information on health effects of EMF during the past year  (information seeking or information scanning)** | | | | | | | |
| neither one nor the other | 77 (5.5) | 1051 (79.4) | 86.1 (86.0-86.3) | 81.8 (80.2-83.0) | 77.1 (75.6-78.5) | 81.7 (78.9-84.6) | 61.3 (58.9-63.6) |
| Only information seeking |  | 88 (6.7) | 4.3 (4.0-4.3) | 5.1 (4.6-5.6) | 6.7 (6.3-7.3) | 6.8 (5.3-8.3) | 16.4 (15.5-17.2) |
| Only information scanning |  | 158 (11.9) | 9.1 (9.1-9.1) | 10.2 (9.4-11.0) | 14.1 (13.0-15.1) | 10.0 (7.3-12.5) | 18.7 (17.0-20.7) |
| Both |  | 26 (2.0) | 0.5 (0.5-0.5) | 2.9 (2.4-3.5) | 2.1 (1.5-2.5) | 1.5 (1.0-2.2) | 3.6 (3.5-3.7) |

S 5: Average Posterior Classification Probability Matix of the LCA

| **Assigned Class** | **LC 1** | **LC 2** | **LC 3** | **LC 4** | **LC 5** |
| --- | --- | --- | --- | --- | --- |
| **Assigned LC 1** | 0.999 | 0.000 | 0.001 | 0.000 | 0.000 |
| **Assigned LC 2** | 0.000 | 0.888 | 0.059 | 0.018 | 0.035 |
| **Assigned LC 3** | 0.000 | 0.070 | 0.918 | 0.000 | 0.011 |
| **Assigned LC 4** | 0.000 | 0.063 | 0.000 | 0.920 | 0.016 |
| **Assigned LC 5** | 0.000 | 0.099 | 0.025 | 0.022 | 0.854 |

S 6: Descriptive values of the variable of subjective information level regarding EMF

| **How well do you feel informed about potential health effects of electromagnetic fields?** |  | **Poorly informed** |  |  |  | **Well informed** |
| --- | --- | --- | --- | --- | --- | --- |
|  | Missing | 0 | 1 | 2 | 3 | 4 |
| Total | 77 (5.5%) | 498 (37.6%) | 330 (24.9%) | 222 (16.8%) | 118 (8.9%) | 155 (11.7%) |
| School teachers | 54 (5.1%) | 357 (35.7%) | 232 (23.2%) | 168 (16.8%) | 96 (9.6%) | 146 (14.6%) |
| Nursery school teachers | 21 (6.4%) | 136 (44.2%) | 93 (30.2%) | 50 (16.2%) | 21 (6.8%) | 8 (2.6%) |

S 7: Sensitivity analysis of the variable regarding the subjective information level, where the middle category of the Likert scale was considered poorly informed; proportion of participants who agreed with the statement; by professional group

| Study population | Empirical | Corrected |
| --- | --- | --- |
| **Total** | 79.4% (77.2%-81.5%) | - |
| **School teachers total** | 75.5% (72.8%-78.2%) | 76.6% (72.4%-79.9%) |
| **Nursery school teachers total** | 90.8% (87.6%-94.1%) | 93.4% (89.9%-95.9%) |

S 8: Prevalence estimates of the outcome variables, including 95% CI; by invitation type of study population

| **Prevalence estimator**  (95%-CI) | **Complete study population**  **(Online + postal)** | | | | **Study population that was invited by post** | | |
| --- | --- | --- | --- | --- | --- | --- | --- |
|  | Total | School teachers | | Nursery school teachers | Total | School teachers | Nursery school teachers |
| **EMF health effects below legal limits (Agreement in %)** | | | | | | | |
| Empirical | 31.0%  (28.5%–33.4%) | 31.2%  (28.4%–34.1%) | | 31.6%  (26.5%–36.6%) | 34.0%  (29.6%–38.5%) | 36.4%  (30.8%–42.1%) | 29.7%  (22.1%–37.3%) |
| Corrected | - | 31.5%  (27.7%–35.6%) | | 33.3%  (27.7%–38.9%) | - | 36.7%  (28.6%–44.9%) | 30.9%  (23.0%–39.9%) |
| **EMF health effects can also have non-physical causes (Agreement in %)** | | | | | | | |
| Empirical | 41.6%  (39.0%–44.2%) | | 41.1%  (38.1%–44.1%) | 42.6%  (37.3%–48.0%) | 47.1%  (42.4%–51.8%) | 46.4%  (40.5%–52.3%) | 46.4%  (38.1%–54.7%) |
| Corrected | - | | 37.6%  (33.2%–42.0%) | 43.7%  (37.8%–50.0%) | - | 45.8%  (39.0%–53.4%) | 47.1%  (37.7%–56.7%) |
| **Relevance of EMF in everyday working life during last 12 months (Proportion of ‘ever relevant’ in %)** | | | | | | | |
| Empirical | 17.6%  (15.6%–19.6%) | | 17.5%  (15.2%–19.9%) | 16.5%  (12.4%–20.5%) | 17.7%  (14.1%–21.4%) | 19.4%  (14.7%-24.1%) | 12.8%  ( 7.1%–18.5%) |
| Corrected | - | | 17.6%  (14.6%–20.6%) | 14.4%  (10.7%–19.0%) |  | 18.9%  (13.6%-24.9%) | 12.3%  ( 7.1%–19.8%) |
| **Poor subjective knowledge regarding EMF (Agreement in %)** | | | | | | | |
| Empirical | 62.6%  (60.0%–65.2%) | | 59.0%  (55.9%–62.1%) | 74.5%  (69.6%–79.4%) | 72.4%  (68.0%–76.7%) | 68.8%  (63.2%-74.4%) | 79.5%  (72.5%–86.5%) |
| Corrected | - | | 56.9%  (52.4%–61.3%) | 76.7%  (71.3%–81.6%) | - | 68.0%  (60.3%–75.0%) | 81.2%  (72.3%–88.2%) |
